# Household food insecurity and its impact on child and adolescent health outcomes in Western high-income countries: a rapid review of mechanisms and associations

**DOI:** 10.1017/S1368980025101092

**Published:** 2025-08-19

**Authors:** Sarah Abraham, Penny Breeze, Hannah Lambie-Mumford, Anthea Sutton

**Affiliations:** 1 University of Sheffield, School of Health and Related Research, Regent Court (ScHARR), 30 Regent Street, Sheffield S1 4DA, UK; 2 University of Sheffield, Department of Politics and International Relations, Modular Teaching Village, Northumberland Road, Sheffield S10 1AJ, UK

**Keywords:** Food insecurity, Child health outcomes, Adolescent health outcomes, Mechanisms, Conceptual framework

## Abstract

**Objective:**

The primary aim of this rapid review was to provide a summary of the mechanisms by which household food insecurity (HFI) is associated with child and adolescent health outcomes. The secondary aim was to identify key HFI determinants, provide an updated account of HFI-associated child/adolescent health outcomes and build a conceptual map to illustrate and consolidate the findings.

**Design:**

A rapid review was performed using EMBASE, Medline, Web of Science and the Cochrane Library. Inclusion criteria were observational high-income English-language studies, studies evaluating the mechanisms and associations between HFI and child health outcomes using statistical methods.

**Setting:**

High-income English-speaking countries.

**Participants:**

Child (3–10 years) and adolescent populations (11–24 years) and their parents, if appropriate.

**Results:**

Eight studies reported on the mechanisms by which HFI is related to child health outcomes, suggesting that maternal mental health and parenting stress play mediating roles between HFI and child/adolescent mental health, behaviour and child weight status. Sixty studies reported on associations between HFI and various child health outcomes. HFI had a significant impact on diet and mental health, which appeared to be interrelated. Sociodemographic factors were identified as determinants of HFI and moderated the relationship between HFI and child/adolescent health outcomes.

**Conclusions:**

There is a gap in the evidence explaining the mechanistic role of diet quality between HFI and child weight status, as well as the interplay between diet, eating behaviours and mental health on physical child health outcomes. The conceptual map highlights opportunities for intervention and policy evaluations using complex systems approaches.

Household food insecurity (HFI) is broadly described as an ‘uncertainty about future food availability and access, insufficiency in the amount and kind of food required for a healthy lifestyle, or the need to use socially unacceptable ways to acquire food’^(1)^. HFI is usually determined by a lack of household financial resources and can have a detrimental impact on the health and well-being of all members of a household, including children.

In 2022, the Food Foundation reported that 4 million children in the UK were experiencing HFI^(2)^, and a previously published rapid review of HFI and child health outcomes found that HFI had a detrimental impact on the physical and psychosocial well-being of children and adolescents^(3)^. There is also evidence to suggest that the harmful health impact of HFI in childhood may have detrimental health consequences into adolescence and early adulthood^(4,5)^. However, little is known about *how* HFI leads to or is associated with poor child and adolescent health outcomes.

To the authors’ knowledge, no review has attempted to synthesise the literature on mechanisms to consider HFI’s multiple health impacts and impacts over time. This rapid review aims to fill this evidence gap and additionally provide an updated rapid review of child/adolescent health outcomes associated with HFI, targeting child and adolescent populations in Western high-income countries (HIC), which reflect the UK child/adolescent population.

## Aims

The primary aim of this review was to identify and summarise the current literature reporting on the mechanisms by which HFI is associated with child and adolescent health outcomes. The secondary aim of this review was to provide an updated account of the key HFI determinants and the child/adolescent health outcomes associated with HFI. The review was used to inform a conceptual model of HFI and child/adolescent health outcomes to illustrate and consolidate the review findings, for the purposes of highlighting areas for intervention and policy planning.

## Methods

### Study design

The aims of this review were met using rapid review methods. The fast-growing academic and policy interest in HFI, coupled with the urgency to synthesise good-quality evidence in a timely manner, meant that rapid review methods were preferred over systematic review methods. Rapid reviews use transparent, systematic review search methods to identify and synthesise evidence, while offering flexibility in their methodology. This study design allowed for streamlined search strategies without compromising validity, which aligned with the exploratory and scoping nature of this review^(6)^. The elements of the systematic review methodology that were adapted for this rapid review were that a grey literature review was not conducted, one reviewer screened and extracted studies and quality assessment was limited to studies reporting on mechanisms only. The lead author was supported by co-authors in shaping the study objectives and search strategy.

### Search strategy

An initial scoping search was performed in PubMed and Google Scholar to identify key publications and retrieve keywords for the search strategy. The search strategy was designed to capture studies that would fulfil both the primary and secondary aims of the review. The search was divided into two concepts: (i) food insecurity/food poverty and (ii) quantitative analysis (encompassing statistical, theoretical and conceptual models relevant to the research problem). An initial scoping search in PubMed found that food insecurity and food poverty were often used interchangeably in studies; thus, both were incorporated as key terms in the search strategy. Searches were conducted in databases: Medline 1946; Web of Science, EMBASE 1947; and the Cochrane Library for articles up to March 2022 (searches were limited to studies published within the past 15 years). Search terms were used as topic headings and Medical Subject Headings and are present in Table 1. Further studies were retrieved using backward and forward citation searching of included articles. The scope of the review was limited to the retrieval of peer-reviewed published literature.

**Table 1:** Search strategy including search concepts, search terms and their combinations

Concept	No	Search statement
Concept 1: Food insecurity	1	Food insecurity/
	2	Food security/
	3	Food insecurity. Ti.
	4	Food security. Ti.
	5	Food poverty. Ti.
	6	1 or 2 or 3 or 4 or 5
Concept 2: Quantitative analysis	7	Statistics as topic/
	8	Structural equation model*. Ti, ab.
	9	Regression. Ti, ab.
	10	Conceptual model*. Ti, ab.
	11	Theoretical model*. Ti, ab.
	12	7 or 8 or 9 or 10 or 11
	13	6 AND 12

* The asterisk denotes a truncation symbol used to capture all word variants beginning with the specified root (e.g., model* retrieves “model,” “models,” “modeling,” “modelled”).

### Inclusion and exclusion criteria

Observational studies were included to reflect the complex relationships between HFI and child/adolescent health outcomes in real-world settings. Studies were included if their population of interest comprised children (aged 3–10 years), adolescents (aged 11–24 years, with a mean age < 19 years) or child/adolescent–parent dyads. The adolescent age range was based on developmental models, which recognised that important aspects of physical, mental and social development continue into early adulthood^(7)^. Younger children (aged under 3 years) were not included in the scope of this review. The health impacts of HFI on children can differ by age group, and infants may be more vulnerable to nutrient deficiencies related to HFI, which may contribute to developmental delays in the early years of life^(8)^.

Studies from Western English-speaking HIC, including the UK, Ireland, the USA, Canada and Australia, were included. Studies from these countries were included to closely generalise the impact of HFI on child/adolescent health outcomes to the UK child/adolescent population. Studies from low- and middle-income countries (LMIC) were excluded due to differences in food systems, nutritional challenges in child/adolescent populations and social policy^(9,10)^. HFI in LMIC may be associated with infectious diseases, such as malaria, which are uncommon in Western HIC^(11)^.

Studies were included if the population exposure was defined as HFI or food poverty. The definition of HFI was decided based on the US Department of Agriculture definition of food insecurity: food insecurity is a ‘household-level economic and social condition of limited or uncertain access to adequate food’^(12)^. Only quantitative studies were included, as the review sought to summarise studies that had used statistical methods to confirm relationships between HFI and health outcomes. An advantage of focusing on quantitative studies was that they have utility beyond this review, such as providing insight into parameters for modelling based on the conceptual model. While qualitative studies provide valuable insight into individuals’ lived experiences of HFI, they were excluded as they do not provide the statistical rigour required to identify population-level correlational relationships between HFI, health outcomes and potential mediators.

Studies were selected based on their reporting of child and adolescent health outcomes. Health outcomes were agreed upon by the authors and discussed for discrepancies. Health outcomes were defined as metabolic risk factors (e.g. BMI/cholesterol/fasting glucose levels), health-related conditions (including physical and psychosocial conditions) and biological processes (e.g. sleep). Further details on inclusion/exclusion criteria are presented in Table 2. The lead author of the paper reviewed titles and abstracts to be included in full-text review, and inclusion was determined by the same reviewer with support from co-authors when examining the full text of publications.

**Table 2:** Inclusion and exclusion criteria used to include studies

Criteria points	Inclusion	Exclusion
Population	Children and adolescents Households with children Child–parent dyads	Non-human studies Adults only studies (> 24 years)
Geography	High-income developed countries	Low-middle-income countries Developing countries that do not reflect the UK child and adolescent population
Study design	Observational studies	Clinical trials Systematic review
Settings	Communities, schools, households	
Exposure of interest	Household food insecurity Food poverty	
Outcome of interest	Impact of exposure on any child/adolescent health outcomes Association between exposure and mediators of non-communicable diseases and conditions Variables identified as mechanisms	
Language	English-language studies	Non-English-language studies
Date of publication	Articles published in the last 15 years	Articles published over 15 years ago
Publication type	Journal articles	Conference papers Supplementary papers Poster presentations

### Data extraction

The Cochrane data collection form was adapted by removing items that were not relevant for this review, such as experimental designs, for example, duration of participation, number of missing participants and intention-to-treat analysis details^(13)^. Variables extracted included sample size, country, datasets used, participant information (e.g. sociodemographic characteristics), HFI measurement tools, the participant reporting HFI, the health outcome of interest, statistical methods and key study results. Data extraction was completed by the lead author.

### Study quality

The Quality in Prognosis Studies (QUIPS) tool analysed the quality of mechanism studies only for risk of bias, as these were the primary studies of this paper^(14)^. Studies scored low, medium or high risk of bias based on an established set of thresholds within the QUIPS tool.

## Results

### Description of included studies

**Figure 1:**
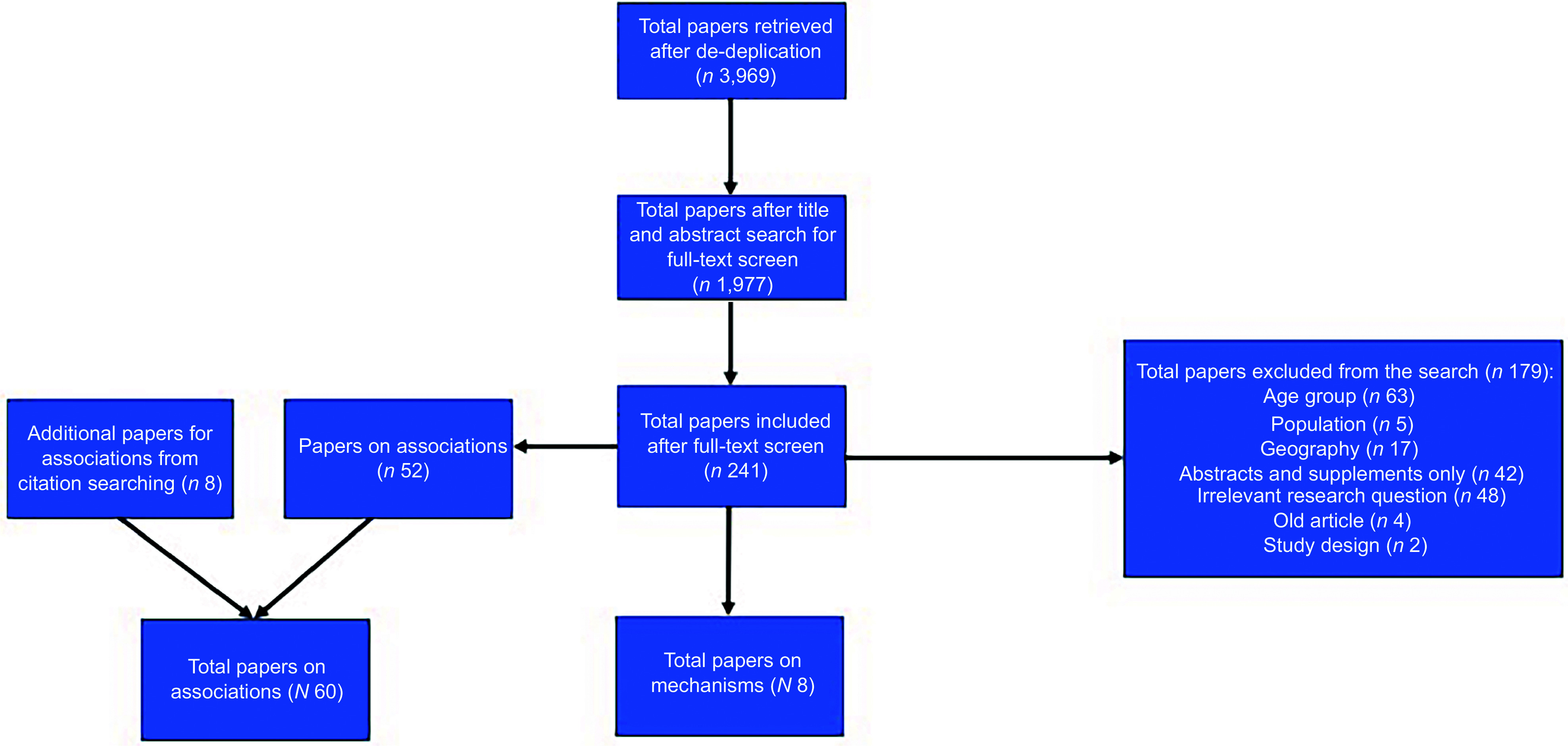
Summary of search results for studies assessing the mechanisms by which household food insecurity (HFI) relates to child and adolescent health outcomes and studies assessing the association of HFI and various child and adolescent health outcomes (including appropriate parental outcomes). *Backward and forward citation searching strategy identified additional studies assessing associations of HFI and child and adolescent health outcomes.

Figure 1 presents a flow diagram of the search results. After removing duplicates, the search results retrieved *n* 1977 articles, and their titles and abstracts were screened. A total of *n* 241 were eligible for full-text screening, of which *n* 8 were mechanism studies^(15–22)^ and *n* 60 were association studies^(23–82)^ (*n* 52 found by the search strategy and an additional *n* 8 retrieved by citation searching strategy).

Most studies included were of cross-sectional design (*n* 59), and the remaining were longitudinal (*n* 10)^(17,19–21,38,40,46,57,62,81,82)^. In terms of geography, *n* 2 studies were based in Australia^(38,70)^, *n* 6 from Canada^(39,50,51,58,62,67)^, *n* 1 from the UK, *n* 1 from Ireland^(65)^ and *n* 59 from the USA. In terms of child age, *n* 8 studies included the child age group (3–10 years old)^(17,27,43,47,49,52,56,81)^, *n* 29 looked at a mix of child and adolescent populations^(15,18,24,28,31,32,38,42,48,50,53,54,57–60,65,70,72–74,77,79,80,82)^ and the rest of the studies (*n* 34) investigated adolescent populations (11–24 years) only.

Some studies investigated more than one health condition; therefore, there is an overlap in the numbers. Of the total studies included in this review, *n* 21 investigated weight status, *n* 3 investigated dental cavities, *n* 2 investigated asthma, *n* 4 on diabetes/prediabetes risk, *n* 5 investigated blood pressure, *n* 4 investigated cholesterol and other metabolic markers of disease, *n* 3 investigated sleep, *n* 3 investigated smoking, drinking and substance abuse, *n* 19 investigated diet, *n* 14 investigated mental health and behaviour, *n* 2 investigated physical activity, *n* 3 investigated quality of life, *n* 10 investigated eating behaviours, *n* 1 investigated anaemia and *n* 1 investigated bone mass disparities.

Studies reporting on mechanisms are presented in Table 3, while studies reporting on associations are presented in Table 4. Studies are presented by health outcome below.

**Table 3:** Included studies that evaluate mechanisms by which HFI may be related to child/adolescent health outcomes

Study	Country	Dataset (design)	Sample size	Child age (years)	Food insecurity Measure (person reporting)	Health outcome	Statistical method	Key results	QA
Bahanan *et al.* (2021)^(15)^	US	NHANES 2011/2012 and 2013/2014 (cross-sectional)	*n* 4822	5–17	USDA module eighteen-item questionnaire of HFI experience over the past 12 months (parent)	Dental cavities Dietary quality	Multiple logistic regression predicting caries from HFI and diet quality. Bivariate analyses Classic mediation methods (Baron and Kenny approach) Confounders included age, sex, race, family income, food programme participation, household size, dental visits and total number of teeth	Untreated dental caries were more likely to occur in food insecure than food-secure children (aOR = 1·38; 95 % CI 1·11, 1·72). Very low HFI (aOR = 1·59; 95 % CU 1·12, 2·26) and marginal HFI (aOR = 1·48, 95 % CI 1·10, 2·01) were associated with untreated caries, but low HFI was not (aOR = 1·20; 95 % CI 0·92, 1·55). Food security was associated with greater whole fruit consumption (*P* = 0·01) and plant protein consumption (*P* = 0·008), but diet quality was not associated with untreated caries (*P* = 0·07).	Low
Do *et al.* (2021)^(16)^	US	FLASHE study (cross-sectional)	*n* 1544	12–17	US Food Security two-item screener (parent)	Obesity/overweight Sleep	Multinomial regression to test for the association between outcomes and HFI Conditional processing modelling to evaluate the moderating and mediating roles of HFI and sleep Cofounders included age, gender, race/ethnicity, parent weight status, parent education and parent income.	HFI was associated with overweight (OR = 1·56; 95 % CI 1·13, 2·15) and obesity (OR = 1·96; 95 % CI 1·40, 2·74; *P* < 0·05). HFI was associated with overweight (aOR = 1·40; 95 % CI 1·00, 2·00; *P* < 0·05). HFI was not associated with mean-centred sleep duration (*β* = –0·08, se = 0·23, *P* = 0·73). Sleep did not mediate the relationship between HFI and weight status HFI did not moderate the relationship between mean-centred sleep duration (*P* = 0·109) and weight status (*P* = 0·206). HFI did not moderate the relationship between sleep and weight status.	Low
Gee and Asim (2019)^(17)^	US	Early Childhood Longitudinal Study-Kindergarten Class of 2010–2011 (longitudinal)	*n* 7820	4–5	First ten items USDA Survey Module (parent)	Parental aggravation Child behaviour, internalising and externalising	First-difference regression analysis to estimate how HFI related to parenting aggravation. Structural equation to test whether parenting aggravation mediated the relationship between HFI (reported 12 months prior to spring of 1st grade) and children’s outcomes (reported in spring of 1st grade) Confounders included: Parent cohabitation status, employment, receipt of food stamps, number of siblings, household socio-economic status, access to medical care, parent–teacher meeting participation.	HFI associated with parental aggravation (*β* = 0·376, se = 0·12, *P* < 0·01) which was indirectly related to children’s lowered ability to pay attention (*β* = –0·062, se = 0·026, *P* < 0·05), inhibitory control (*β* = –0·093, se = 0·027, *P* < 0·001), externalising behaviours (*β* = 0·43, se = 0·12, *P* < 0·001) but not internalising behaviours (*β* = 0·008, se = 0·10). No direct paths from HFI to these child health outcomes were observed.	Low
Gundersen *et al.* (2008)^(18)^	US	NHANES 1999–2002 (cross-sectional)	*n* 841	3–17	USDA module eighteen-item questionnaire of HFI experience over the past 12 months (parent)	Obesity/overweight Four maternal stress indices (cumulative mental stressor index, cumulative physical stressor index, cumulative family structure stressor index, total cumulative stressor index).	Probit maximum likelihood models used to assess associations with a child’s probability of being at risk of overweight. Five models estimated: 1. HFI 2. HFI and four maternal stressor indices 3. HFI, the four maternal stressors and the interaction of these indices with HFI 4. HFI and a total cumulative maternal stressor index 5. HFI, the total cumulative maternal stressor index and the interaction of this index with HFI Confounders included: child age, income-to-poverty ratio, child ethnicity, maternal BMI, maternal age and maternal education.	HFI alone was not associated with child overweight/obesity (*β* = –0·15; 95 % CI –0·38, 0·09). The impact of maternal stressors on child overweight is reduced when the household is food insecure (*β* = 0·05; 95 % CI 0·00, 0·10). When stratified by age, an association between cumulative maternal stressors and HFI was found for children aged 3–10 years old, which was linked to being overweight (*β* = 0·08; 95 % CI 0·02, 0·15; *P* < 0·05). The effect was not seen in children aged 11–17.	Low
Hatem *et al.* (2020)^(19)^	US	Fragile Families and Child Well-Being Study (longitudinal)	*n* 2626	15	USDA module eighteen-item questionnaire of HFI experience over the past 12 months (parent)	Adolescent mental health Maternal depression and stress	Structural equation modelling Confounders included: child gender, health insurance, child race/ethnicity, maternal and household characteristics at year 3: education, cohabitation, employment, income (based on the family income to the federal poverty line) and parent incarceration.	HFI at age 5 had a direct relationship with adolescent anxiety symptoms (*β* = 0·913; 95 % CI 0·064, 1·814; *P* < 0·001). An indirect effect from HFI and housing instability at age 5 was observed via maternal depression to parenting stress to adolescent anxiety (*β* = 0·055; 95 % CI 0·007, 0·110). A direct association was observed for HFI at age 5 and greater depressive symptoms in adolescence (*β* = 0·626; 95 % CI 0·052, 1·226). An indirect effect was observed for housing instability and HFI via parenting stress to maternal depression to adolescent depression (*β* = 0·008; 95 % CI 0·002, 0·016). Maternal depression predicted higher parenting stress (*β* = 0·161; 95 % CI 0·096, 0·227).	Moderate
Lohman *et al.* (2009)^(20)^	US	Welfare, Children and Families: A three-city study – a study of families below 200 % of the poverty line (longitudinal)	*n* 1011	10–15	USDA module eighteen-item questionnaire of HFI experience over the past 12 months (parent)	Overweight/obesity Individual/maternal/family stressors (individual stressor index, maternal stressor index and family stressor index)	Logistic regression modelling, two models: 1. HFI and the stressor indices 2. HFI, the stressor indices and the interaction of these indices with HFI Confounders included: Age, gender, ethnicity, television viewing, disability limitations and low birth weight. Maternal immigration status, in receipt of food stamps, education, BMI and age. Household’s income-to-needs ratio.	Maternal stressors interact with HFI, and both are associated with child overweight/obesity, and this was statistically significant (OR = 1·79; 95 % CI 0·82, 3·92; *P* < 0·001).	Low
Marcal (2022)^(21)^	US	Fragile families and child well-being longitudinal survey (longitudinal)	*n* 2454	15	USDA module eighteen-item questionnaire of HFI experience over the past 12 months (parent)	Adolescent mental health Parenting stress	Structural equation modelling Confounders included: Maternal age, ethnicity, education, household income, cohabitation status, family history of mental disorder and child gender.	HFI reported at age 5 years was not directly associated with greater adolescent depressive behaviours (*β* = –0·109; 95 % CI –0·253, 0·002) or aggressive behaviours (*β* = –0·031; 95 % CI –0·134, 0·058). HFI reported at age 5 years was indirectly associated with adolescent aggressive behaviours (*β* = 0·025; 95 % CI 0·009, 0·049) and depressive behaviours (*β* = 0·032; 95 % CI 0·0132, 0·064) via parenting stress reported when the child was aged 9 years.	Low
Willis and Fitzpatrick (2016)^(22)^	US	Data from a school in Northwest Arkansas in 2012 (cross-sectional)	*n* 324	10–13	USDA module eighteen-item questionnaire of HFI experience over the past 12 months (parent)	Weight status	Ordinary least squares regression Confounders included: gender and ethnicity	HFI was associated with weight status (*P* < 0·001). This relationship was mediated by social status (*β* = –0·112; *P* < 0·05) and depression (*β* = 0·109; *P* < 0·05).	Moderate

aOR, adjusted OR; HFI, household food insecurity; QA, quality assessment. USDA, US Department of Agriculture.

**Table 4:** Included studies that evaluate associations between HFI and various child/adolescent health outcomes

Study	Country	Dataset (design)	Sample size	Child age (years)	Food insecurity measure (person reporting)	Health outcome	Statistical method	Key results
Altman *et al.* (2019)^(23)^	US	N/A data obtained for analysis from a randomised trial (trial)	*n* 4822	10–14	Child Food Security Assessment over the prior 12 months (child)	Mental health Self-esteem/body dissatisfaction	Multivariable logistic regression Confounders included: Race/ethnicity, free/reduced priced school meals (proxy for socio-economic status), age and gender.	Food insecure adolescents had greater odds of reporting body dissatisfaction, and this was prevalent across children of all ethnic groups and BMI categories. The association was stronger in children of normal weight (OR = 1·76, *P* < 0·001) and of African American ethnicity (OR = 2·32, *P* < 0·001).
Appelhans *et al.* (2014)^(24)^	US	Home Environment Comparison Study (cross-sectional)	*n* 103	6–13	USDA module eighteen-item questionnaire of HFI experience over the past 12 months (parent)	Food availability Child eating behaviours	Logistic regression Confounders included: Negative cooking attitudes, financial strain, household size, caregiver employment status and education	Increased food security was associated with increased availability of home food supplies (*P* = 0·02). HFI was associated with more frequent family meals (very low HFI (OR = 1·95; 95 % CI 0·55, 6·81), low HFI (OR = 3·44; 95 % CI 1·29, 9·18), and home-made dinners (very low HFI (OR = 1·51; 95 % CI 0·34, 6·84), low HFI (OR = 1.07; 95% CI= 0.35-3.24)).
Bauer *et al.* (2015)^(25)^	US	EAT 2010 and Project F-EAT (cross-sectional)	*n* 2807	14	Six-item US Household Food Security Survey Module assessment of HFI experience over the past 12 months (parent)	Weight status (obesity) Parental feeding styles	*χ* ^2^ tests and F-tests Confounders included: Mothers’ BMI, household income, number of children living in the house (under 18 years old), mothers’ education, employment status, race/ethnicity, adolescent age and gender	Association found between HFI severity and BMI percentiles in males (food-secure mean (sd) = 65·8 (0·3), low food security mean (sd) = 72·0 (0·30), very low food security mean (sd) = 70·01 (0·30) (*P* = 0·02)
Bruening *et al.* (2017)^(26)^	US	Exploratory pilot study recruitment from six public housing sites 2014 (cross-sectional)	*n* 55	12–16	Six-item US Household Food Security Survey Module assessment of HFI experience over the past 12 months (parent)	Mother eating habits and behaviours. Child eating habits and behaviours	Multivariate mixed linear Logistic regression Confounders included: Maternal age, US-born status, cohabitation status, education status, household, child age and gender	Adolescent intake of fast food (*P* < 0·001), binge eating (*P* = 0·011), intuitive eating (*P* = 0·032) and partaking in family meals (*P* = 0·001) was significantly associated with mothers after. The odds of these associations between mother and child were greater in food insecure households.
Canter, Roberts and Davis (2017)^(27)^	US	Recruitment while receiving services or through other organisations providing services to low-income parents/caregivers 2013 (cross-sectional)	*n* 148	5–10	USDA module eighteen-item questionnaire of HFI experience over the past 12 months (parent)	Dietary quality Physical activity	Structural equation modelling Confounders included: Child gender, race/ethnicity, annual income, household size, children in the household (under 18 years)	HFI was significantly associated with lower consumption of vegetables (*P* < 0·05) Children who did more physical activity were more likely to consume more vegetables (*P* < 0·01).
Drucker *et al.* (2019)^(28)^	US	The Midlands Family Study (cross-sectional)	*n* 511	< 18	USDA module eighteen-item questionnaire of HFI experience over the past 12 months (parent)	Parental characteristics: caregiver life experience	Multinomial logistic regression Confounders included: Child age, gender, ethnicity, household income	HFI was significantly associated with negative caregiver life experiences (OR = 1·16, 95 % CI 0·88, 1·04, *P* < 0·05), which is in turn was associated with greater odds of child hunger (OR = 1·08; 95 % CI 1·05, 1·11; *P* < 0·05)
Duke (2021)^(29)^	US	2019 Minnesota Student Survey (cross-sectional)	*n* 125 375	12–19	One question on the survey: ‘During the past 30 d, have you had to skip meals because your family did not have enough money to buy food?’ (adolescent)	Prediabetes risk BMI/weight status	Multivariable logistic regression Confounders: Low quality diet, physical activity, BMI, sleep, age, gender, socio-economic indicator (free or reduced-price lunch), region, ethnicity	HFI was associated with prediabetes (OR = 1·93; 95 % CI 1·59, 2·32; *P* < 0·001). Significant associations highest between HFI and prediabetes were observed for non-Hispanic Black African Americans (OR = 1·88; 95 % CI 1·52, 2·31; *P* < 0·001), Hispanic/Latino (OR = 2·49; 95 % CI 2·06, 3·01; *P* < 0·001) and non-Hispanic White students (OR = 2·20; 95 % CI 1·49, 3·25; *P* < 0·001) BMI strong predictor of prediabetes (OR = 4·34; 95 % CI 3·84, 4·90; *P* < 0·001)
Duke (2021)^(30)^	US	2019 Minnesota Student Survey (cross-sectional)	*n* 125 375	12–19	One question on the survey: ‘During the past 30 d, have you had to skip meals because your family did not have enough money to buy food?’ (adolescent)	Dietary intake and quality Eating behaviour	Multivariable logistic regression Model 1: association between HFI and food/beverage consumption accounting for sociodemographic covariates and housing instability Model 2 multivariable association between HFI and food/beverage consumption accounting for sociodemographic covariates, housing instability and indicators of physical and emotional health Confounders included: Gender, age, race/ethnicity, free or reduced-price lunch, region, housing instability	Results are from model 2 but are similar to model 1 results as well. Food insecure adolescents were more likely to consume less than the minimum recommended intake for fruit (OR = 0·76; 95 % CI 0·69, 0·82; *P* < 0·001), dairy products (OR = 0·67; 95 % CI 0·58 = 0·76; *P* < 0·001) and vegetables (OR = 0·86; 95 % CI 0·75, 0·98; *P* < 0·001) than those who are food-secure Food insecure adolescents were more likely to consume more sugar-sweetened beverages (OR = 1·21; 95 % CI 1·12, 1·30; *P* < 0·001) and fast-food items (OR = 1·62; 95 % CI 1·49, 1·76; *P* < 0·001) than food-secure adolescents Food insecure adolescents had greater odds of skipping lunch than food-secure adolescents (OR = 1·80; 95 % CI 1·67, 1·95; *P* < 0·001).
Dykstra *et al.* (2016)^(31)^	US	Part of a larger survey of convenience sample of mothers (cross-sectional)	*n* 821	2–13	Six-item US Household Food Security Survey Module assessment of HFI experience over the past 12 months (parent)	Dietary intake and quality	Linear regression– accounting for within school clustering of outcomes Confounders included: Child age, gender, free or reduced-price lunch status, ethnicity	HFI was associated with reduced dairy product consumption food insecure 37·8 % *v*. 49·4 % in food-secure children, *P* < 0·03) HFI associated with increased candy consumption (11·5 % *v*. 8·4 %) than food-secure children, *P* < 0·03.
Eicher-Miller *et al.* (2011)^(32)^	US	NHANES 1999–2004 (cross-sectional)	*n* 11 247	12–15	USDA module eighteen-item questionnaire of HFI experience over the past 12 months (parent if child < 11)	Iron deficiency. Anaemia	Logistic regression Confounders included: Poverty-to-income ratio, race ethnicity, smoker in the house, birth weight, age, gender, use of dietary supplements, household participant in welfare or food assistance programmes	HFI associated with 2·95 times greater odds of iron deficiency anaemia (OR = 2·95; 95 % CI 1·18, 7·37; *P* = 0·02).
Eicher-Miller *et al.* (2009)^(33)^	US	NHANES 2001–2004 (cross-sectional)	*n* 5270	8–19	USDA module eighteen-item questionnaire of HFI experience over the past 12 months (parent if child < 11)	Dietary intake Micronutrient deficiency Bone mass disparities	Multiple linear regression for bone mass content content (gender specific) Multiple logistic regression for micronutrient analysis (genders together) Confounders included: Survey cycle year, race/ethnicity, poverty income ratio, smoking, use of dietary supplement, participation in welfare or food assistance programmes	HFI was associated with reduced odds of consuming the USDA food guide recommended serving of dairy products (OR = 2·5; 95 % CI 1·1, 5·8; *P* = 0·03) in male adolescents HFI was associated with reduced odds of consuming recommended calcium intake (OR = 2·3; 95 % CI 1·3, 4·0; *P* < 0·01) HFI was associated with less bone mineral content in the total body (*P* = 0·04), spine (*P* = 0·02), left arm (*P* = 0·05) and trunk (*P* = 0·05) in males aged 8–11.
Fleming *et al.* (2021)^(34)^	US	NHANES 2007–2016 (cross-sectional)	*n* 4777	13–18	Household: USDA module eighteen-item questionnaire of HFI experience over the past 12 months (parent)	Weight status, obesity	Multivariable logistic regression Confounders included: Age, race, sex, household poverty level, routine access to healthcare	There was a higher prevalence obesity in food insecure than food-secure child populations (PR = 1·3; 95 % CI 1·2, 1·5; *P* < 0·001). HFI was not statistically significantly associated with obesity (OR = 1·19; 95 % CI 1·0, 1·4; *P* = 0·08).
Fram *et al.* (2015)^(35)^	US	NHANES 2007–2016 (cross-sectional)	*n* 3605	12–17	USDA module eighteen-item questionnaire of HFI experience over the past 12 months (parent)	Dietary intake Adolescent awareness of HFI	Mixed-effects linear and logistic regression Alpha 0·05 for significance and alpha < 0·10 considered marginal significance Confounders included: child age, gender and intervention group	HFI was associated with higher consumption of energy (OR = 49·61; 95 % CI 9·80, 89·43; *P* = 0·02), fat, (OR = 0·45; 95 % CI 0·013, 0·89; *P* = 0·05), sugar (OR = 0·79; 95 % CI 0·0098, 1·57; *P* = 0·05) and fibre (*β* = 0·13; 95 % CI 0·023, 0·24; *P* = 0·02). HFI was associated with reduced consumption of vegetables (*β* = –1·52; 95 % CI –4·11, 1·08; *P* = 0·25) and increased consumption of fruit (*β* = 1·36; 95 % CI –2·44, 5·16; *P* = 0·48), but this was NS. Adolescents showed awareness of HFI in terms of their cognition, emotions and physical (hunger) recognition.
Fulay *et al.* (2022)^(36)^	US	NHANES 2007–2016 (cross-sectional)	*n* 5076	12–17	USDA module eighteen-item questionnaire of HFI experience over the past 12 months (parent	Child weight status BMI Metabolic biomarkers (glucose, TAG, cholesterol) Blood pressure	Multivariable linear regression Confounders included: Age, sex, race/ethnicity, activity level, household income: poverty, respondent education level and marital status	No significant association was determined between HFI and child BMI (*β* = 0·14; 95 % CI –0·01, 0·28), systolic blood pressure (*β* = 0·18; 95 % CI –1·07, 1·43, diastolic blood pressure (*β* = –0·09; 95 % CI –1·25, 1·07) and metabolic biomarkers (including total cholesterol (*β* = –1·07; 95 % CI –4·69-2·55, fasting TAG (*β* = 3·81; 95 % CI –4·61 –12·24) and fasting blood plasma glucose (*β* = 0·97; 95 % CI = –0·52, 2·47) All models adjusted for age, ethnicity, gender, smoking, sedentary time, activity, respondent education, marital status and income All *P*-values > 0·05
Fulkerson *et al.* (2009)^(37)^	US	Team COOL (cross-sectional)	*n* 145	15–18	One-item assessment in survey (adolescent): ‘How often during the past 12 months have you been hungry because your family couldn’t afford more food?’	Dietary intake Child mental health Eating behaviour	Mixed model logistic and linear regression Confounders included: Race/ethnicity, SES < age, gender	Adolescents who did not participate in family meals had greater odds of being overweight (OR = 2·8; 95 % CI 1·1, 6·9; *P* < 0·05) and food insecure (OR = 6·0; 95 % CI 2·2, 16·4; *P* < 0·05) than children who are family meals. Family meals were associated with food security, increase breakfast (*P* < 0·05) and fruit consumption (*P* < 0·01) as well as lower rates of depressive symptoms in adolescents (*P* < 0·05)
Gasser *et al.* (2017)^(38)^	Australia	Longitudinal study of Australian Children (longitudinal)	*n* 4569–5107	2–15	One item on the questionnaire asked to parent ‘whether over the last 12 months, due to shortage of money, they had financial limits on the type of food they could buy’	Dietary intake/quality	Multinomial logistic regression Confounders included: Childs number of older siblings, child from multiple births or not, parental education, socio-economic status, age, ethnicity, parents’ age	Poorer children tended to report higher on the ‘never healthy’ rather than ‘always healthy’ domain compared with children of higher socio-economic groups (OR = 16·40; 95 % CI 9·40, 28·61; *P* < 0·05).
Godrich *et al.* (2019)^(39)^	Canada	Canadian Children’s Lifestyle and School Performance Study II (cross-sectional)	*n* 5281	10–11	Six-item US Household Food Security Survey Module assessment of HFI experience over the past 12 months (parent)	Child mental health (self-esteem)	Multivariable logistic regression Confounders included: Gender, region of residence, parent educational attainment, household income and bodyweight status	HFI was associated with higher odds of poor self-esteem insecure in girls (OR = 2·19; 95 % CI 1·57, 3·07; *P* < 0·001) and severely food insecure boys (OR = 1·68; 95 % CI 1·11, 2·53; *P* = 0·014)
Gundersen *et al.* (2008)^(40)^	US	The Three City Study (longitudinal)	*n* 1031	10–15	Three items taken from child-specific questions of the USDA module eighteen-item questionnaire of HFI experience over the past 12 months (parent).	Obesity/overweight/BMI	Logistic regression Confounders included: Child ethnicity, race/ethnicity. Household: needs ratio caregiver education level; immigration level, whether the family eat meals together, housing stability, caregiver marital status, caregiver age and household size	HFI was associated with overweight, but this was not a statistically significant difference observed (OR = 0·97; 95 % CI 0·65, 1·86; *P* = 0·72)
Gundersen *et al.* (2009)^(41)^	US	NHANES 2001–2004 (cross-sectional)	*n* 2516	8–17	USDA module eighteen-item questionnaire of HFI experience over the past 12 months (parent)	Obesity measures (BMI, waist circumference, triceps skinfold, trunk fat mass and percentage of body fat	Logistic regression Confounders included: child age, race/ethnicity, gender, annual household income:poverty	No statistically significant association reported. For the risk of obesity: BMI and food insecure (OR = 1·14; 95 % CI 0·84, 1·54) Weight circumference and food insecure (OR = 1·136; 95 % CI 0·848, 1·523) Triceps skinfold and HFI (OR = 1·070; 95 % CI 0·754, 1·519) Trunk fat mass and HFI (OR = 1·21; 95 % CI 0·858, 1·465) Body fat % and HFI (OR = 1·051; 95 % CI 0·821, 1·346) No *P*-values provided in this article.
Hill (2019)^(42)^	US	NHANES 2013/2014 (cross-sectional)	*n* 4406	1–19	USDA module eighteen-item questionnaire of HFI experience over the past 12 months (parent)	Dental cavities	Logistic regression Confounders included: Race/ethnicity, age, income: poverty, assistance programmes, dental caries status	Children from very low food-secure households were more likely to have dental caries than food-secure children (OR = 3·51; 95 % CI 1·71, 7·19; *P* = 0·0003) Odds were greater in older age groups (*P* < 0·0001)
Hobbs and King (2018)^(43)^	US	Fragile Families and Child Well-Being Longitudinal Study (cross-sectional)	*n* 2046	5	USDA module eighteen-item questionnaire of HFI experience over the past 12 months (parent)	Child mental health and behaviour problems	Unconditional quantile regression Confounders included: Mothers race/ethnicity, low birth weight, maternal education mother cohabitation status, mothers’ employment status, parent smoking and drug use, mother immigration status, assistance programme	HFI was associated with more behavioural problems (externalising and internalising), and the association was greater in children with the most behaviour problems (*P* < 0·05)
Holben and Taylor (2015)^(44)^	US	NHANES 1999–2006 (cross-sectional)	*n* 7435	12–18	USDA module eighteen-item questionnaire of HFI experience over the past 12 months (parent)	Obesity/overweight/BMI Blood pressure Cholesterol, TAG, HDL-cholesterol, LDL-cholesterol	Logistic regression Confounders included: age, sex and ethnicity	Children who were marginally food-secure, low food-secure and very low food-secure were 1·3–1·4 times more likely to be overweight than children experiencing high food security (*P* = 0·036) Children who were marginally food-secure were 1·2–1·4 times more likely to be obese than children who were fully food-secure (*P* = 0·036) Marginally, low and very low food security was associated with 1·4 to 1·5 times greater central obesity than high food security (*P* = 0·002). There was no statistically significant difference in mean blood pressure across levels of HFI (high *v*. marginal OR = 0·83, 95 % CI 0·54, 1·28; high *v*. low OR = 0·99, 95 % CI 0·54, 1·81; high *v*. very low OR = 1·49, 95 % CI 0·94, 2·34) (*P* = 0·31) There was no difference in mean glucose (*P* = 0·59) and TAG levels (*P* = 0·97) between food security severities.
Hooper *et al.* (2020)^(45)^	US	EAT and Project F-EAT (cross-sectional)	*n* 2285	10–22	Short form six-item USDA-based form of the past 12 months (parent)	Obesity/overweight/BMI Child eating behaviour	Logistic and negative binomial regression Analyses performed separately between males and females Confounders included: Age, gender ethnicity/race, parental education	The prevalence of obesity among food insecure populations was higher than in food-secure (42·3 % *v*. 39·8 %; *P* = 0·039) In analyses, adjusting for gender, ethnicity, age and parental education HFI was not associated with overweight (35·6 % *v*. 37·6 %, *P* = 0·39). FI adolescents reported lower breakfast consumption per week (*P* = 0·005) FI adolescents partook in more unhealthy weight-control behaviours (fasting < 0·001, eating very little food *P* = 0·006, skipping meals *P* = 0·001, laxative use *P* = 0·018, diuretic use 0·001) compared with food-secure adolescents (*P* < 0·001)
Jackson and Vaughn (2017)^(46)^	US	Early Childhood Longitudinal Study, Kindergarten class 1998–1999 (longitudinal)	*n* 7028	13–14	USDA module eighteen-item questionnaire of HFI experience over the past 12 months (parent)	Child mental health, misconduct behaviours	Logistic regression Confounders included: Age, race/ethnicity BMI, neurological defects, SES composite score (parental education, employment, occupational prestige, income, neighbourhood disadvantage), low parental involvement, physical punishment and parent depression	Persistent HFI was associated with misconduct disorder in males (OR = 1·73; 95 % CI 1·44, 2·07) Misconduct rate was 96 % higher in persistently food insecure males than food-secure males Misconduct rate was 39 % higher in persistently food insecure females than food-secure females
Jackson and Testa (2021)^(47)^	US	National Survey of Children’s Health 2016–2018 (cross-sectional)	*n* 99 962	9–10	One question on a cross-sectional survey with multiple choice answers: ‘Which of these statements best described the food situation in your household in the past 12 months?’	Oral health	Logistic regression and negative binomial regression, four models: (1) unadjusted models for each child oral health outcome, (2) adjusting for covariate, (3) further adjustments for socio-economic, insurance and oral health care variables	Moderate-Severe HFI was associated with poorer oral health across all models (model 3 results OR = 1·91; 95 % CI 1·61, 2·27; *P* < 0·01) and tooth decay/dental cavities (model 3 results OR = 1·56; 95 % CI 1·38, 1·75; *P* < 0·01).
Kaur *et al.* (2015)^(48)^	US	NHANES 2001–2010 (cross-sectional)	*n* 9701	2–11	USDA module eighteen-item questionnaire of HFI experience over the past 12 months (parent)	Obesity	Logistic regression Confounders included: Sex, ethnicity, poverty-to-income ratio and survey period	HFI was associated with obesity in children aged 6–11 (OR = 1·81; 95 % CI 1·33, 2·48; *P* < 0·001) but not in younger children aged 2–5 years old (OR = 0·88; 95 % CI 0·51, 1·51; *P* = 0·64). Models were adjusted for sex, ethnicity, poverty-to-income ratio and survey period.
King (2017)^(49)^	US	Fragile Families and Child Well-Being Study (cross-sectional)	*n* 2829	5	USDA module eighteen-item questionnaire of HFI experience over the past 12 months (parent)	Sleep SSB consumption	Multivariate ordinary least squares regression Confounders included: Gender, maternal education, income: poverty, ethnicity, number of children in the household and parent relationship status	HFI associated with increased consumption of soft drinks compared with food-secure children (51·4 % *v*. 41·2 %, *P* < 0·001). HFI associated with trouble sleeping at night compared with food-secure children (30·1 % *v*. 25 %, *P* < 0·001)
Kirk *et al.* (2015)^(51)^	Canada	The Children’s Lifestyle and School Performance Study (cross-sectional)	*n* 5853	10–11	Short form six-item version of the US Household Food Security Survey Module	Obesity/overweight Dietary intake/quality Quality of life Child mental health	Linear and Poisson regression models with robust standard errors Confounders included: Parent education, area of residence, gender	Marginal, moderate and severe HFI were associated with greater BMI z-scores, when models adjusted for gender and area of residence. Children who are food insecure are less likely to meet recommended fruit and vegetable (PR = 0·73–0·83, *P* < 0·05) and dairy products (0·83, 0·86, *P* < 0·05) targets from Canada’s Food Guide Moderate or severe food insecurity associated with higher energy intake(*P* < 0·05) HFI is associated with poorer quality of life (measured using EQ5D). high food security *v*. moderate HFI (*β* = –0·02; 95 % CI –0·04, –0·01; *P* < 0·05) high food security *v*. severe HFI (*β* = –0·03; 95 % CI –0·04, 0·01). Models adjusted for gender and area of residence.
Kirkpatrick and Tarasuk (2008)^(50)^	Canada	Canadian Community Health Survey (cross-sectional)	*n* 3348	1–18	USDA module eighteen-item questionnaire of HFI experience over the past 12 months (parent)	Dietary intake	ANOVA Confounders included: Household income, household education level (adults and children), immigration status, household size (number of adults and children), smokers	HFI was not associated with fruit and vegetable servings in children aged 4–8 years old (*P* = 0·16). HFI associated with servings of grains in 4–8 years old (0·0) HFI was associated with nutrient deficiencies in vitamin B_6_, vitamin B_12_, Zn, vitamin A, phosphorus, vitamin A and Mg among older children and adolescents. HFI was associated with increased energy consumption (*P* = 0·09) and less energy consumed from protein products (*P* < 0·01)
Kral, Chittams and Moore (2017)^(52)^	US	Cross-sectional analysis part of larger laboratory-based feeding study (cross-sectional)	*n* 50	8–10	Short form six-item US Household Food Security Survey Module assessment of HFI experience over the past 12 months (parent)	Parent feeding style and child eating behaviour	Wilcoxon rank-sum tests *t* tests *χ* ^2^ and Fisher’s test. Logistic regression Confounders included: Mother’s race/ethnicity, age, education, household income, child meal and snack patterns, frequency of mother telling child to clear their plate	Children who were obese were more likely to be food insecure than food-secure (OR = 4·9; 95 % CI 1·15, 20·8; *P* = 0·02). Food insecure children were more likely to overeat when they were not hungry (*P* < 0·03) and consume more snacks than children who were food-secure (45·9 % *v*. 15·4 %, *P* = 0·02) Food insecure children were more likely to consume 5 or more snacks than per day than food-secure children (15·4 % *v*. 0 %, *P* = 0·02)
Kuku *et al.* (2011)^(53)^	US	Child Development Supplement data of larger dataset Panel Study of Income Dynamics (cross-sectional)	*n* 959	0–12	USDA module eighteen-item questionnaire of HFI experience over the past 12 months (parent)	Obesity	Parameter probit regression Non-parameter regression Confounders included: Race/ethnicity, gender, income: poverty	No association was observed between HFI and obesity in parametric probit regression In non-parametric analysis, the probability of obesity rises as HFI increases, except at extreme HFI where it lowers. Higher levels of HFI are associated with obesity in girls, in boys’ lower levels of HFI and an increase in HFI affirmative responses is associated with a higher probability of obesity. The association between HFI severity and obesity differs among ethnic groups.
Landry *et al.* (2019)^(54)^	US	TX Sprouts (cross-sectional)	*n* 598	8–11	USDA module eighteen-item questionnaire of HFI experience over the past 12 months (parent)	Dietary intake and quality	Mixed-effects linear regression Confounders included: Race/ethnicity, gender, participation in food assistance programmes, average energy intake and BMI percentile	HFI was associated with lower consumption of green beans (2·3 % *v*. 1·9 %, *P* = 0·016) plant protein and seafood (2·0 % *v*. 1·6 %, *P* = 0·006) and increased consumption of added sugar (7·4 % *v*. 8 %, *P* = 0·002) compared with food-secure children.
Lee *et al.* (2019)^(62)^	US	NHANES 2003–2014 (cross-sectional)	*n* 2662	12–19	USDA module eighteen-item questionnaire of HFI experience over the past 12 months (parent)	Blood pressure Prediabetes	Logistic regression Confounders included: Age, gender, income, race/ethnicity, household income: poverty	Food insecure adolescents were more likely to have hypertension (OR = 1·65; 95 % CI 1·38, 2·1·98; *P* < 0·05) and be diagnosed as prediabetic (OR = 1·96; 95 % CI 1·17,-3·19; *P* < 0·05) compared with food-secure adolescents
Mangini *et al.* (2015)^(56)^	US	Early Childhood Longitudinal Study-Kindergarten Cohort (cross-sectional)	*n* 1109	8–9	USDA module eighteen-item questionnaire of HFI experience over the past 12 months (parent)	Asthma	Multivariate logistic regression Analysed overall and by race/ethnicity Confounders included: Race/ethnicity, household poverty: income, child gender, maternal nativity, maternal education, child health insurance, height, weight and household income	HFI was associated with higher odds of asthma (OR = 1·04; 95 % CI 1·02, 1·06; *P* < 0·001) Food insecure children had double the odds of reporting asthma compared with food-secure children who were wealthier (OR = 2·00; 95 % CI 1·97, 2·03; *P* < 0·001)
Mangini *et al.* (2019)^(57)^	US	Early Childhood Longitudinal Study-Kindergarten Cohort (longitudinal)	*n* 6731	4–14	USDA module eighteen-item questionnaire of HFI experience over the past 12 months (parent)	Asthma	Two multiple logistic regression models: (1) estimation of OR by asthma diagnosis at third grade exposure to HFI in year prior to kindergarten, then exposure in the year prior to kindergarten and third grade, (2) estimation of OR of asthma diagnosis at fifth or eighth grade by exposure to HFI in second grade Confounders included: Household poverty, race/ethnicity, sex, health insurance coverage, BMI and maternal nativity and parental depression	Children who experienced HFI the year before joining kindergarten had greater odds of an asthma diagnosis in the third grade (OR = 1·18; 95 % CI 1·17, 1·20; *P* < 0·001) Children who experience food insecurity in the year before third grade are more likely to be diagnosed with asthma compared with food-secure children (OR = 1·53; 95 % CI 1·51, 1·55; *P* < 0·001) Children were more likely to have an asthma diagnosis from third to eighth grade if their parents experienced depression before they joined kindergarten (OR = 1·23; 95 % CI 1·22, 1·25; *P* < 0·001)
Marjerrison *et al.* (2011)^(58)^	Canada	Two general paediatric practices from Nova Scotia (cross-sectional)	*n* 183 families	7–16	USDA module eighteen-item questionnaire of HFI experience over the past 12 months (parent)	Diabetes Hospitalisation	Backwards stepwise elimination, logistic regression Confounders included: Parent education, household income, child age, number of other family members with medical conditions	HFI was associated with greater HBA1c concentration (9·5 % ± 2·13 % *v*. 8·96 % ± 1·50 %, *P* = 0·039, but this was insignificant when child’s age and parents’ education in multivariate analysis. HFI was associated with higher rates of hospitalisation (OR = 3·66; 95 % CI 1·54, 8·66; *P* < 0·05).
Martin and Ferris (2007)^(59)^	US	Convenience sample (cross-sectional)	*n* 200 parents and *n* 212 children	2–12	USDA module eighteen-item questionnaire of HFI experience over the past 12 months (parent)	Obesity/overweight	Multinomial regression Confounders included: Income below 100 % of poverty, child age, gender, parent cohabitation status, parent education status	Children with family incomes below 100 % poverty line are half as likely to be overweight as those with higher incomes (OR = 0·47, *P* = 0·05) HFI was associated with overweight, but this was not statistically significant (OR = 1·41; 95 % CI 0·67, 2·99; *P* = 0·37) Female gender and having an obese parent were associated with greater odds of child overweight (OR = 2·56, *P* = 0·01)
Masler *et al.* (2020)^(60)^	US	NHANES 2005–2012 (cross-sectional)	*n* 6077	8–15	USDA module eighteen-item questionnaire of HFI experience over the past 12 months (parent)	Child eating behaviours	Multivariate logistic regression, separate models for (1) HFI and weight loss attempts, (2) HFI and weight-control practices Confounders included: Age, sex, race/ethnicity, poverty status and weight class	No association was found between overweight/obese children, food insecurity and unhealthy weight loss behaviours (*P*-values range 0·75, 0·98, 0·12 for marginal food-secure, low food-secure and very low food-secure, respectively. Very low food security and healthy weight is associated with greater odds of attempted weight loss (OR = 1·51; 95 % CI 1·00, 2·26; *P* < 0·05) and unhealthy weight control in children (OR = 1·42; 95 % CI 1·04, 1·93; *P* < 0·05)
Maynard *et al.* (2019)^(61)^	US	NHANES 2009–2010 (cross-sectional)	*n* 935	12–17	USDA module eighteen-item questionnaire of HFI experience over the past 12 months (parent)	Child mental health/anxiety	Logistic regression examined the interaction between HFI and sex/gender separately, as this was statistically significant, the model was repeated stratified by sex. Confounders included: Race/ethnicity, household size, parental education, family poverty:income ratio, child gender	HFI associated with greater odds of experiencing high levels of anxiety compared with (OR = 1·64; 95 % CI 1·04, 2·60) food-secure children, considering both genders. Female gender (OR = 1·85; 95 % CI 1·18, 2·91) and increasing age (OR = 1·28; 95 % 1·16, 1·43) were associated with higher levels of anxiety In the stratified model, food insecure females had greater odds of high anxiety (OR = 0·3·03; 95 % CI 1·59, 5·80), but relationship was not observed in male gendered children. *P*-values were not reported, but significance was reported by the author.
McIntyre *et al.* (2013)^(62)^	Canada	Canadian National Longitudinal Survey of Children and Youth 1994–2008/2009 (longitudinal)	*n* 22 831	13–17	One question to assess child hunger (adolescent)	Child mental health, depression and suicide	Logistic regression. All analyses were weighted using longitudinal weights. No interaction terms found nor included in the analyses. Confounders included: Child age, gender, housing stability, lived with biological mother, lived with alone mother, depression, household education, household income, household size	Food insecure adolescents who reported hunger were more likely to experience depression/suicide ideation (OR = 2·3; 95 % CI 1·2, 4·3; *P* = 0·006)
McLaughlin *et al.* (2012)^(63)^	US	National comorbidity survey replication adolescent supplement (cross-sectional)	*n* 6483 adolescent–parent dyads	13–17	Two dichotomous questions about hunger over the past 12 months (parent)	Child mental health	Logistic regression Confounders included: Parent education, household income, relative deprivation and subjective social status, age, gender, race/ethnicity	HFI was associated with increased odds of anxiety (OR = 1·1; 95 % CI 1·0, 1·2), behaviour disorder (OR = 1·2; 95 % CI 1·1, 1·3), substance disorders (OR = 1·2; 95 % CI 1·0, 1·3) and mood disorders (OR = 1·1; 95 % CI 1·0, 1·2). All *P*-values < 0·05 significant
Mendoza *et al.* (2018)^(64)^	US	SEARCH for Diabetes in Youth (cross-sectional)	*n* 226	10–20 (mean age 15·6)	USDA module eighteen-item questionnaire of HFI experience over the past 12 months (parent)	Diabetes Hospitalisations	Logistic regression for high-risk glycaemic control Binomial regression for emergency department visits and hospitalisations Confounders included: Age, sex, race/ethnicity, socio-economic status, study site, diabetes duration, health insurance and insulin regimen.	HFI was associated with greater odds of increased HBA1c (OR = 2·37; 95 % CI 1·10, 1·59; *P* < 0·05) HFI was associated with greater emergency department visits (PR = 2·95; 95 % CI 1·17, 7·45; *P* = 0·02) HFI was not statistically significantly associated with hospitalisations but had a higher prevalence than in food-secure youths (PR = 2·96; 95 % CI 0·92, 9·51; *P* = 0·07).
Molcho *et al.* (2007)^(65)^	Ireland	Health Behaviour in School-aged Children study (cross-sectional)	*n* 8424	10–17	Food poverty question in survey: ‘Some young people go to school or to bed hungry because there is not enough food at home. How often does this happen to you?’	Dietary intake Eating behaviour	Logistic regressing modelling Confounders included: Age and social class (according to fathers’ education)	Food poverty significantly associated with consuming less fruit and vegetables and wholemeal bread (OR range 0·66–0·81, 95 % CI range 0·45–0·99) Food poverty was associated with increased consumption of crisps, fried potato and hamburger (OR range 1·29–1·72, 95 % CI range 1·00–1·85) Increased consumption of fast food differed among girls and boys Food poverty was associated with reduced breakfast intake within the week for food insecure girls, OR =1·72 (95 % CI 1·50, 1·9) and food insecure boys OR = 1·29 f(95 % CI 0·99, 1·59) All values are significant at *P* < 0·05.
Niemeier and Fitzpatrick (2019)^(66)^	US	Sample of high school students from Northwest Arkansas (cross-sectional)	*n* 1493	13–16	Household: USDA module eighteen-item questionnaire of HFI experience over the past 12 months (parent)	Obesity/overweight Mental health Self-esteem	Ordinal regression using the Polytomous Universal Model Confounders included: Gender, ethnicity, parents’ immigration status, free and reduced lunches	HFI was associated with poor self-esteem (*P* < 0·01), less frequent family meals and weaker relationships with peers (*P* < 0·001) No association was found between HFI and weight status
Ovenell *et al.* (2022)^(67)^	Canada	Canadian Community Health Survey (cross-sectional)	*n* 28 871	12–17	Household: USDA module eighteen-item questionnaire of HFI experience over the past 12 months (parent)	Child mental health	Poisson regression Confounders included: Gender, race/ethnicity, household size, household income, highest household education, high BMI, survey cycle	Children who experienced food insecurity and were not shielded by its impacts had elevated risk of anxiety (RR = 1·44, 95 % CI 1·02, 2·03) and poor mental health (RR = 1·45; 95 % CI 1·06, 1·99) and poor life satisfaction (RR = 1·64; 95 % CI 1·15, 2·35) Children who were shielded from HFI had a higher risk of anxiety (RR = 1·45, 95 % CI 1·02, 3·03 and fair or poor mental health (OR = 1·56; 95 % CI 2·07, 2·09) and did not differ significantly from food-secure children with respect to other mental health outcomes. All significant findings: *P* < 0·05.
Parker *et al.* (2010)^(68)^	US	NHANES 1999–2006 (cross-sectional)	*n* 3126	12–19 (mean age 15)	USDA module eighteen-item questionnaire of HFI experience over the past 12 months (parent)	Obesity/overweight Physical activity Blood pressure Metabolic syndrome (indicated by cholesterol, TAG	Logistic regression Confounders included: Age, sex, race/ethnicity, household income, smoking status, highest household education	Food-secure children were more likely to report doing vigorous exercise compared with food insecure children (*P* < 0·05). HFI (marginal, low and very low) was not associated with metabolic syndrome. Model 1 results adjusted for ethnicity, age and gender: Marginal (OR = 1·04; 95 % CI 0·38, 2·86), low = (OR = 1·49; 95 % CI 0·79, 2·82), very low (OR = 1·18; 95 % CI 0·47, 2·95). Model 2 results adjusted for by ethnicity, age, gender, income, smoking status and education: Marginal (OR = 0·94; 95 % CI 0·35, 2·51), low (OR = 1·12; 95 % CI 9·57, 2·17) and very low (OR = 0·89; 95 % CI 0·35, 2·23). No statistically significant association was found in the prevalence of HFI severity, including blood pressure and TAG, glucose and HDL-cholesterol across food insecurity severities (*P* > 0·05). There was no association between HFI and metabolic syndrome across HFI severities when using full food security as reference (full *v*. marginal OR = 1·04, 95 % CI 0·38, 2·86, full *v*. low OR = 1·49, 95 % CI 0·79, 2·82, full *v*. very low OR = 1·18, 95 % CI 0·47, 2·95) after adjusting for ethnicity, sex and age. Similar results observed when adding covariates household income, smoking level and education
Poulsen *et al.* (2019)^(69)^	US	Electronic health record data (cross-sectional)	*n* 408 parent-child dyads	10–15	Six-item USDA-based form of the past 12 months (parent reported)	Obesity/overweight Dietary intake Food availability	Multivariable linear regression Confounders included: child age, child sex, child race/ethnicity, parent age, family income, parent education and youth history of medical assistance	In unadjusted analyses HFI associated with greater mean BMI z-scores (*β* = 0·33; 95 % CI 0·06, 0·61; *P* = 0·018), waist circumferences (*β* = 0·26; 95 % CI 0·04, 0·48; *P* = 0·027) and % body fat (*β* = 0·45; 95 % CI 0·15, 0·75; *P* = 0·020) In analyses adjusted for age, gender, ethnicity, parental age and income, HFI was associated with greater waist circumference (*β* = 0·27; 95 % CI 0·03, 0·50; *P* = 0·02), % body fat ((*β* = 0·43; 95 % CI 0·12, 0·75; *P* = 0·006) but not BMI z-scores (*β* = 0·30; 95 % CI –0·00, 0·60, 0·051) Children from homes with low food availability had reduced fruit and vegetable intake (*β* = 0·08; se = 0·02; *P* = 0·003) HFI was not associated with daily fruit and vegetable consumption in adjusted analyses (*β* = –0·06; 95 % CI –0·56, 0·43; *P* = 0·805)
Ramsey *et al.* (2011)^(70)^	Australia	Census collector districts, Brisbane Statistical Sub-Vision (cross-sectional)	*n* 185 households	3–17	USDA module sixteen-item questionnaire of HFI experience over the past 12 months (parent)	Child mental health and behaviour	Multinomial logistic regression Confounders included: Household income, education, employment status, household structure (how many dependents and parents marital status)	HFI was associated with negative internalised emotional symptoms such as depression (OR = 2·44; 95 % CI 1·11, 5·38; *P* = 0·05) Food insecure children and adolescents were also more likely to exhibit problem behaviour, but this was borderline statistically insignificant (OR = 2·35; 95 % CI 1·04, 5·33; *P* = 0·06) and also present conduct problems (OR = 1·69; 95 % CI 0·84, 2·47; *P* = 0·03).
Robson *et al.* (2017)^(71)^	US	Youth Risk Behaviour survey (cross-sectional)	*n* 4994	12–18	Single item on the survey ‘During the past 30 d, how often did you go hungry because there was not enough food in your home?’ (adolescent)	Obesity and overweight Sleep Cigarette smoking Alcohol consumption Dietary intake Eating behaviour	Univariate logistic regression Confounders included: Race/ethnicity, child age, child gender, neighbourhood safety, school grade	HFI was associated with reduced consumption of breakfast throughout the week (OR = 2·27; 95 % CI 1·6, 3·21; *P* < 0·001) HFI was associated with sleeping less than 8 h per day (OR = 1·60; 95 % CI 1·15, 2·23; *P* = 0·006) HFI was statistically significantly associated with smoking (OR = 1·81; 95 % CI 1·31, 2·40; *P* < 0·001) and drinking (OR = 1·39, 95 % CI 1·07, 1·80; *P* = 0·01) No significant association between HFI and obesity was found (OR = 1·107; 95 % CI 0·82, 1·4; *P* = 0·61)
Soldavini and Ammerman (2021)^(72)^	US	USDA Summer Electronic Benefit Transfer for Children Demonstration Project (cross-sectional)	*n* 11 873	3–17	USDA module eighteen-item questionnaire of HFI experience over the past 12 months (parent)	Dietary intake	Multiple linear regression Confounders included: Child gender, caregiver race/ethnicity, caregiver education, household income: poverty threshold, household composition, household size, number of children in the household, participation in food assistance programmes	Marginal, low and very low food security were associated with lower consumption of fruits and vegetables. Very low food security was associated with lowest consumption of fruit and veg compared with food-secure children across all age groups (*P* < 0·001). Children aged 5–8 experiencing low food security consumed less wholegrains than food-secure children (B = –0·16; 95 % CI –0·31, –0·00; *P* < 0·05). Children aged 13–17 experiencing very low food security consumed less wholegrains than food-secure children (B = –0·42; 95 % CI –0·83, 0·01; *P* < 0·05). Children aged 5–8 from marginally food-secure households consumed more added sugar (B = 0·97; 95 % CI 0·03, 1·91; *P* < 0·05), this was also observed in 3–4-year-old children who were low food-secure (B = –2·85, 95 % CI –4·50, –1·20, *P* < 0·001). Low food-secure 3–5-year-olds consumed more sugar from sugar-sweetened beverages compared with food-secure children (B = –1·84, 95 % CI –3·15, 0·54, *P* < 0·05). All food insecure children consumed less dairy products than food-secure children (*P* < 0·001)
South *et al.* (2019)^(73)^	US	NHANES 2007–2014 (cross-sectional)	*n* 7125	8–17 (mean age 12)	USDA module eighteen-item questionnaire of HFI experience over the past 12 months (parent)	Blood pressure	Multivariable logistic regression Confounders included: BMI, race/ethnicity, child age, child gender, household income	HFI was associated with high blood pressure (OR = 1·26; 95 % CI 1·04, 1·54; *P* < 0·05)
Tan *et al.* (2019)^(74)^	US	Children’s PowerPlay! Campaign (cross-sectional)	*n* 3547	9–11	Five questions from the Child Food Security Assessment (child)	Dietary intake	Multivariable mixed-effects linear and logistic regression Confounders included: Gender, ethnicity/race, poverty level and survey year.	HFI in girls was associated with an increase in total kilocalories consumed (135 calories per day, *P* < 0·005) and 60 calories from snacks per day at the highest severity of FI (*P* < 0·05) compared with girls who were food-secure The same relationship was not seen for male children. This study found no association between HFI and meal patterns, including missing meals for either gender.
Tester *et al.* (2016)^(75)^	US	NHANES 2003–2010 (cross-sectional)	*n* 1072	12–18	USDA module eighteen-item questionnaire of HFI experience over the past 12 months (parent)	Fasting TC, LDL-cholesterol, TAG, HDL-cholesterol, TAG/HDL-cholesterol ratio and (Apo B)	Logistic regression Confounders included: Race/ethnicity, household income, household size, poverty income ratio, parent cohabiting, maternal education	Marginal HFI was associated with elevated TAG (OR = 1·86; 95 % CI 1·14, 3·06), TAG/HDL-cholesterol (OR = 1·74; 95 % CI 1·11, 2·82) and Apo B (OR = 1·98; 95 % CI 1·17, 3·36). Marginally food-secure females had lower HDL-cholesterol than males (OR = 2·69; 95 % CI 1·14, 6·37). *P*-value not reported but significance stated.
Tevie and Shaya (2018)^(76)^	US	NHANES 2005–2012 (cross-sectional)	*n* 6334	12–18	USDA module eighteen-item questionnaire of HFI experience over the past 12 months (parent)	Child mental health	Multivariate logistic regression for three models (1) a cut-off for poor mental health days of < 14 d considered baseline, (2) cut-off of < 7 d and (3) a cut-off of < 4 d. Confounders included: Poverty: income, gender, race/ethnicity, child age, child gender, parent education level.	Adolescents who experienced very low food security were more likely to suffer from bad mental health estimated by model 1 (OR = 2·06; 95 % CI 1·63, 2·58; *P* < 0·0001), model 2 (OR = 1·98; 95 % CI 1·67, 2·29; *P* < 0·0001) and model 3 (OR = 1·94; 95 % CI 1·14, 3·28; *P* = 0·01) compared with adolescents from food-secure households.
To *et al.* (2014)^(69)^	US	NHANES 2003–2006 (Cross-sectional)	*n* 3049	6–17	USDA module eighteen-item questionnaire of HFI experience over the past 12 months (parent)	Physical activity	Linear regression for identifying associations between HFI and time spent doing physical activity or being sedentary Confounders included: Age, gender, race/ethnicity, parent marital status, education, household size and household income	HFI was associated with less participation in moderate-to-vigorous physical activity (*β* = –5·24, *P* = 0·02), but there was no association between HFI and sedentary minutes (*β* = 8·87, *P* = 0·07).
Turner *et al.* (2022)^(78)^	US	Youth Risk Behaviour Survey 2017 (cross-sectional)	*n* 43 857	14–19	One question: ‘During the past 30 d, how often did you go hungry because there was not enough food in your house?’ (adolescent)	Substance abuse Cigarette smoking Alcohol consumption	Bivariate analyses using *χ* ^2^ tests. Logistic regression calculated for adjusted prevalent ratios (PR) Confounders included: Gender, age, ethnicity, school grade, sexual identity, current binge drinking, current marijuana use	Results from bivariate analyses: Food insecure adolescents were more likely to participate in binge drinking, abuse of illegal substances such as marijuana as well as prescription drugs (*P* < 0·05) Results from multivariable logistic regression: HFI was also associated a lifetime usage of prescription opioid substance abuse (PR = 1·38; 95 % CI 1·12, 1·71; *P* = 0·005) and use of illicit drugs (PR = 1·70; 95 % CI 1·22, 2·37; *P* = 0·003).
Widome *et al.* (2009)^(79)^	US	Project EAT 1998–1999 class (cross-sectional)	*n* 4746	< 18	Two items adapted from the USDA hunger core food security module HFI experience over the past 12 months (parent)	Dietary intake Eating behaviour	Multiple linear regression Confounders included: Age, grade level, gender, race/ethnicity, family income	HFI was associated with greater consumption of fat (*P* < 0·01) and ate fewer family meals (*P* < 0·001) and breakfast (*P* = 0·001) HFI was associated with less food availability of unhealthy and healthy food in the household (*P* < 0·001) across all hunger frequencies. Both food-secure and food insecure understood the benefits of healthy eating, across all hunger frequencies.
Wirth *et al.* (2020)^(80)^	US	Chart review of children visiting clinic (cross-sectional)	*n* 2688	2–17	HVS screening measure binary rating system over the past 12 months (child)	Overweight/obesity	Logistic regression Confounders included: Race/ethnicity, gender, insurance type, weight status, tobacco exposure, household composition, neighbourhood poverty rate	Food insecure children and adolescents were less likely to be obese compared with food-secure (RRR = 0·56; 95 % CI 0·36, 0·87) HFI was negatively associated with obesity (OR = 0·55; 95 % CI 0·35, 0·86; *P* < 0·01).
Yang *et al.* (2018)^(81)^	UK	Born in Bradford study (longitudinal)	*n* 1101 children	4–5	USDA module eighteen-item questionnaire of HFI experience over the past 12 months (parent)	BMI z-score Dietary intake	Quantile and logistic regression- each ethnic group (White British and Pakistani analysed separately) Confounders included: Mothers’ ethnicity, maternal age, maternal cohabitation status, maternal education, household size, subjective financial insecurity, IMD.	White British mothers were more likely to report HFI than Pakistani mothers (11 % *v*. 7 %, *P* < 0·01). From 12 months of age to 4–5 years old, prevalence of overweight and obesity increased both among food-secure (10–22 %) and food insecure (8–22 %) Pakistani-origin children. In regression models: BMI z-score increased by 0·25 units (95 % CI 0·20, 0·29) for Pakistani-origin children who were food-secure and 0·40 units (95 % CI 0·22, 0·59) for their food insecure counterparts. Among White British children the prevalence of overweight increased among food-secure children (12–25 %) but a decrease was observed among food insecure children (24–19 %). In White British children, greater increases were observed for BMI z-scores among food-secure children (*β* = 0·17; 95 % CI 0·13, 0·21). This was statistically significant. In White British food insecure children, a smaller increase in BMI z-scores was observed (*β* = 0·06; 95 % CI –0·05, 0·17). Pakistani mothers were more likely to live in the most deprived areas or receive means-tested benefits compared with White British mothers.
Zhu *et al.* (2019)^(82)^	US	Early Childhood Longitudinal Study-Kindergarten Cohort 1998–2007 (longitudinal)		8–14	USDA module eighteen-item questionnaire of HFI experience over the past 12 months (parent)	Obesity/overweight	Logistic regression with Bonferroni adjustment Confounders included: Child gender, race/ethnicity, birth weight, health insurance, household poverty (at/above or below the poverty threshold), parent depression, maternal nativity and maternal education.	Children who experienced HFI in 5th grade had higher BMI z-scores in 5th (OR = 0·19; 95 % CI 0·07, 0·30; *P* < 0·05) and in the 8th grade (OR = 0·17; 95 % CI 0·06, 0·27; *P* < 0·05) Children who were food insecure had a higher risk of overweight/obesity in 3rd, 5th and 8th grade compared with food-secure children (*P* < 0·05).

Apo B, apo B-100; B, beta; PR, prevalence ratio; RR, relative risk.; RRR, relative risk ratio; SES, socioeconomic status; SSB, sugar-sweetened beverages.

### Outcomes

#### Obesity and overweight

Twenty-one studies investigated the relationship between HFI and child overweight/obesity and found mixed results^(16,18,20,22,25,34,36,40,41,44,45,51,53,59,66,68,69,71,80–82)^. Eight studies found no direct association between HFI and child overweight/obesity using various markers of weight status, including BMI, waist circumference, trunk fat mass, triceps skin folds, body fat percentage, metabolic syndrome, BMI z-scores and weight status^(16,36,40,41,59,66,68,71)^. Eleven studies found a statistically significant association between HFI and greater odds of overweight/obesity OR range: 1·44–1·81 (95 % CI 1·13, 2·48)^(25,34,44,45,48,51,53,69,80–82)^. One study only found an association between greater BMI and HFI in adolescent boys^(25)^, while one study found an association in older children and not younger children (< 6 years)^(48)^. Two studies in this review did not report a statistically significant association between HFI and overweight. However, they did find a greater prevalence of obesity in food insecure populations^(34,45)^. Only one study found an association between HFI and reduced odds of obesity^(82)^.

Studies found that the relationship between HFI and child overweight/obesity was determined by child age, child ethnicity, parental education, household income, maternal BMI and severity and timing of HFI^(25,44,51,53,66,68,69,80–82)^. For example, children who were food insecure, older, non-White, whose parents had lower educational attainment level and whose parents were obese were more likely to be overweight. One study found that food insecure girls were more likely to be overweight, compared with food-secure girls, but the same comparison was not drawn for boys^(59)^. Furthermore, persistent HFI and timing of HFI were also associated with both weight extremes. Children who experienced persistent HFI throughout their life from early childhood to early adolescence had higher odds of being overweight, while children who experienced a single exposure to HFI in early childhood were associated with underweight in early adolescence^(53,80,82)^.

Four studies explored the mechanism by which HFI may be associated with weight status^(16,18,20,22)^. Two studies exploring mechanisms found that an interaction between HFI and maternal stressors had an impact on weight status, for children aged 3–10 years old and adolescents aged 10–15 years old; however, results were mixed^(18,20)^. Both studies did not find a direct association between HFI and child overweight. One study found that an interaction between HFI and maternal stressors amplified the probability of food-secure children being obese or overweight, compared with children living in food insecure households, whose mothers experienced similar stressor levels (*P* < 0·05)^(18)^. In contrast, the second study found that maternal stressors enhanced the likelihood of living with overweight or obesity when an adolescent was exposed to HFI (*P* < 0·001)^(20)^.

One study found that while HFI was directly associated with adolescent overweight (OR = 1·56; 95 % CI 1·13, 2·15; *P* < 0·05) and obesity (OR = 1·96; 95 % CI 1·40, 2·74; *P* < 0·05) an indirect relationship was not mediated by sleep duration (*P* = 0·23)^(16)^. Additionally, another study found a direct and indirect relationship between HFI and child overweight, mediated by child psychosocial factors including depression and social status (*P* < 0·05)^(22)^.

#### Diet quality

Nineteen studies reported and found an association between HFI and diet quality in both children and adolescents, using various diet quality indicators^(26,27,30–32,35,38,49–52,54,65,69,71,72,74,79,81)^. Six studies found that food insecure children and adolescents were less likely to consume vegetables compared with their food-secure peers^(27,35,50,54,65,72)^. Three studies found that HFI was associated with reduced consumption of fruit^(51,65,72)^. Four studies in older children (> 10 years) found no association between HFI and fruit and/or vegetable consumption^(35,50,69,71)^. HFI was also associated with reduced healthy and unhealthy food availability in the household^(79)^.

Five studies found that food insecure children and adolescents were less likely to consume dairy products than food-secure children/adolescents^(31,32,50,72)^. Studies also found that children and adolescents were likely to consume greater savoury and sweet snacks, sugar-sweetened beverages and fast foods than those who were food-secure^(30,31,49,52,54,65,72,81)^. Moreover, food insecure children and adolescents were more likely to have higher daily energy intake, with lower energy consumption from protein and wholegrains^(35,50,51,54,74,79)^. Additionally, two studies found that HFI was associated with a reduced intake of vitamins A, B_6_, B_12_, thiamine, Fe, riboflavin, Mg, phosphorus and Zn^(32,50)^. The relationship between HFI and diet quality differed by gender, ethnicity and child age^(32,81)^.

#### Eating behaviours

Ten studies found that HFI was associated with poor eating habits in children and adolescents, characterised by reduced frequency of family meals, meal skipping, binge eating and overeating in the absence of hunger^(24,26,30,37,45,52,60,65,71,79)^. Maternal binge eating and breakfast skipping were associated with food insecure adolescents mimicking this behaviour^(26)^. A US study also found that food insecure children enrolled in a national school lunch programme were more likely to skip lunch than those enrolled in the programme^(30)^. Two studies found that HFI was related to weight-control eating behaviour, including fasting, laxative use and increased weight loss attempts in children and adolescents^(45,60)^.

#### Dental cavities

Three studies found an association between HFI and dental caries, which varied depending on HFI severity and child age ^(15,42,47)^. The odds of dental caries were significantly greater (OR = 3·51; 95 % CI 1·71, 7·19; *P* < 0·05) in children who experienced severe HFI compared with fully food-secure adolescents. Older food insecure children had greater odds of dental caries^(42)^. Children who experienced any HFI across all severities had poorer oral health compared with fully food-secure children^(47)^.

One study investigating mechanisms found that HFI was directly associated with untreated dental caries in children and adolescents (OR = 1·38; 95 % CI 1·11, 1·72; *P* < 0·01). In this study, diet quality was not found to be associated with untreated caries, so the authors did not perform a mediation analysis^(15)^. However, HFI was associated with poorer diet quality in this study.

#### Prediabetes risk

Four studies found evidence demonstrating an association between HFI and increased prediabetes risk^(29,55,58,64)^. HFI was associated with greater HbA1c concentrations (> 9 %, which is high risk) in all studies. Studies reported racial disparities between HFI and prediabetes risk, with higher Hispanic and Black children having greater prediabetes risk (*P* < 0·001) than food insecure children of other races^(55)^. Another study found higher odds of prediabetes risk among food insecure non-White Hispanic adolescents (adjusted ^(64)^ OR (aOR) = 2·83; 95 % CI 2·14, 3·73) compared with food insecure adolescents who were of Black race (aOR = 1·88; 95 % CI 1·12, 3·14) and Hispanic adolescents (aOR = 1·84; 95 % CI 1·14, 2·97)^(29)^.

#### Hospitalisation risk

Two studies found that food insecure children had greater rates of hospitalisation than food-secure^(58)^. HFI was associated with hospitalisations (aOR = 3·66; 95 % CI 1·54, 8·66)^(58)^ and emergency department visits (prevalence ratio = 2·95; 95 % CI 1·17, 7·45)^(64)^.

#### Blood pressure

Five studies reported mixed evidence for the association of HFI with blood pressure^(36,44,55,68,73)^. Three studies found a small positive association, varying by HFI severity, gender, age, ethnicity and household income^(55,68,73)^. Two studies found little or no association^(36,44)^.

#### Cholesterol, fasting glucose and other metabolic markers of health

Three studies found no association between HFI and various metabolic markers of health^(36,44,68)^. One study found a significant difference only for marginally food insecure groups, where the odds of having elevated serum TAG (OR = 1·86 95 %; CI 1·14, 2·82), TAG/HDL-cholesterol (OR = 1·74; 95 % CI 1·11, 2·82) and apo B (OR = 1·98; 95 % CI 1·17, 3·36) were greater than those observed in food-secure groups. In this study, marginally food-secure females had greater odds than males of having low HDL-cholesterol (OR = 2·69; 95 % CI 1·14, 6·37)^(75)^

#### Asthma

Two studies found an association between HFI and asthma, which varied by race, household income and timing of HFI^(56,57)^. Food insecurity was associated with greater odds of asthma in non-Hispanic whites and Hispanics. However, odds were lower in non-Hispanic Black children^(56)^. One study found that the timing of HFI was an important determinant in the association between HFI and asthma diagnosis^(57)^. For example, children who experienced HFI a year before entering kindergarten had 13 % greater odds of an asthma diagnosis in the third grade (OR = 1·13; 95 % CI 1·17, 1·20), while HFI experienced in the year prior to joining third grade was associated with 53 % greater odds of developing asthma in the third grade (95 % CI 1·51, 1·55)^(57)^.

#### Anaemia

One study found a positive association between HFI and anaemia. The odds of iron deficiency anaemia among food insecure adolescents were greater than food-secure adolescents (OR = 2·95; 95 % CI 1·18, 7·37; *P* = 0·02)^(33)^.

#### Bone mass disparities

One US study found HFI to be associated with less bone mass, particularly in food insecure male children, who had significantly lower estimated total body (*P* = 0·05), trunk (*P* = 0·05), spine (*P* = 0·2), pelvis (*P* = 0·05) and left arm (*P* = 0·02) bone mineral content than food-secure males^(32)^. Food insecure males consumed fewer dairy products, thus having lower calcium intake than recommended. HFI was more prevalent in non-Hispanic Black, Mexican American and other ethnic groups.

#### Physical activity

Two studies found that fully food-secure adolescents were more likely to participate in moderate-to-vigorous physical activity than food insecure adolescents (*P* < 0·02)^(68,77)^.

#### Quality of life

Three studies found that food poverty or HFI were significantly associated with lower quality of life^(51)^ and lower life satisfaction^(65,67)^ in children and adolescents aged 10–17 years. Moderate-to-severe HFI was associated with lower health-related quality of life outcomes, particularly psychosocial outcomes (*P* < 0·05)^(51)^.

#### Sleep

Two studies found a negative association between HFI and sleep (*P* < 0·001)^(49,71)^. In both studies, food insecure children and adolescents reported poor sleep quality. One study found that HFI was not associated with mean-centred sleep duration in adolescents^(16)^.

#### Smoking, alcohol and substance abuse

HFI was associated with greater odds of cigarette smoking^(71)^, alcohol consumption^(71)^, opioid misuse and lifetime use of illicit drug use^(63,78)^. The relationship between HFI and smoking, alcohol and substance abuse was dependent on age and ethnicity; for example, one study found that food insecure non-Hispanic Black and non-Hispanic white children of older age were more likely to partake in substance abuse behaviour^(78)^.

#### Mental health and behaviour

Fourteen studies found an association between HFI and detrimental mental health and behavioural difficulties^(17,19,21,23,39,43,46,61–63,66,67,70,76)^. Most studies explored the relationship in older children or adolescents, with only two studies^(17,43)^ reporting in children under 10 years. Four studies found an association between HFI and anxiety, which was significantly worse in females, older children and adolescents and worsened with HFI severity^(61,63,67,76)^. Reports of anxiety were greater in children and adolescents who were not shielded from HFI by caregivers^(67)^. Four studies found an association between HFI and increased depression/atypical emotional symptoms^(62,63,67,70)^. Furthermore, hunger was associated with greater odds of depression and suicidal ideation in adolescents (OR = 2·3; 95 % CI 1·2, 4·3)^(62)^.

Four studies found a positive association between HFI and misconduct, behavioural difficulties and internalising and externalising symptoms in children and adolescents^(43,46,63,70)^. Persistent HFI was associated with greater misconduct in adolescents (bullying/fighting/stealing/cheating/lying/misbehaving), and this was worse in food insecure males than females compared with their food-secure counterparts^(46)^. Additionally, three studies found that HFI was associated with poor self-esteem in older children and adolescents, and this was stronger in girls than in boys ^(23,39,66)^. One study found a positive association between HFI and body dissatisfaction in US adolescents across all races and BMI categories, which was stronger among those with African American race/ethnicity (*P* < 0·001)^(23)^.

Three studies explored mediatory pathways between HFI and child/adolescent mental health and behavioural outcomes. One study found that HFI, reported by the parent in early childhood (aged 5 years), was directly associated with adolescent anxiety and depressive symptoms. The study found that both HFI and housing instability combined had an indirect impact on adolescent anxiety and depression via parenting stress and maternal depression, reported when the child was aged 9 years^(19)^. A second study found that HFI, at age 5, was not directly associated with adolescent aggressive or depressive behaviour; however, it did have an indirect impact on aggressive behaviour and depressive behaviour via parenting stress reported when the child was aged 9 years^(21)^. A third study found that HFI was indirectly associated with lowered ability to pay attention, lower inhibitory control and greater externalising behaviours via parental aggravation in children aged 4–5 years^(17)^.

#### Quality assessment of mechanism studies

Eight mechanism studies, all using data from the USA, in children and adolescents aged 3–17 years, were assessed by the QUIPS tool^(15–17,19–22)^. Low risk of bias was found in seven^(15,16,18,20,21)^ out of eight studies, while moderate bias was found in one^(12)^. Quality assessments are located in the online supplementary material, Supplemental material.

#### Conceptual model of results

This review shows that the evidence relating HFI to health outcomes remains mixed. HFI has detrimental health impacts on child health outcomes for prediabetes risk, dental cavities, bone mass, asthma, anaemia, physical activity, quality of life, behaviour, diet quality and mental health. However, it is unclear whether or how HFI is related to child overweight/obesity, blood pressure and cholesterol/other biomarkers.

**Figure 2:**
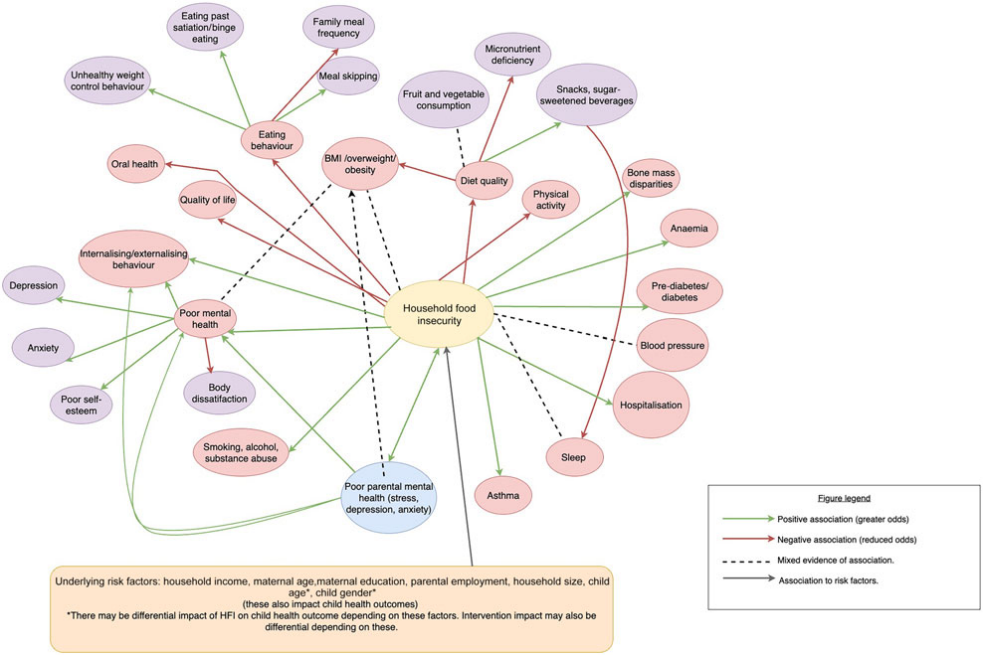
Conceptual framework of review results of mechanisms and associations reported between household food insecurity (HFI) and child/adolescent health outcomes and parental mental health outcomes. Red arrows indicate OR > 1 between HFI and outcomes, green arrows indicate OR > 1 between HFI and outcomes and a thick dashed line indicates mixed evidence regarding an association between HFI and outcomes or between outcomes.

The associations and mechanisms found in this review are illustrated in Fig. 2. The green arrows represent an OR > 1, while the red arrows represent an OR < 1. Dashed lines represent mixed evidence.

## Discussion

This review identified two key mechanistic pathways between HFI and detrimental child/adolescent health outcomes: (i) diet and (ii) mental health, which appeared to be interrelated in complex ways. There was a strength of evidence supporting the role of parent mental health as a mediator between HFI and greater child/adolescent mental health symptoms and behavioural difficulties^(17,19,21)^. One explanation of this mechanism may be that HFI contributes to poor caregiver mental health, which results in parents’ reduced abilities to partake in positive parenting practices and provide parental warmth^(83)^. This mechanism of action aligns with the Family Stress Model, which suggests that financial strain leads to economic pressures (e.g. HFI), which can contribute to caregiver psychological distress and compromised parenting practices that impact child outcomes^(84)^.

This review found mixed evidence for an association between HFI and child weight status, depending on a range of factors, including timing, severity of HFI and sociodemographic factors^(25,44,51,69,81,82)^. While one study found that maternal stressors enhanced the association between HFI and child overweight, another found that the association was enhanced for food-secure children^(18,20)^. Although no association between HFI and overweight was concluded, there is evidence of higher obesity prevalence among food insecure populations^(34,45)^. These findings are echoed in the literature, which has described this as the food insecurity–obesity paradox, which has been explained by a multitude of factors, including that individuals may eat more when food is in abundance and reduce their intake when food availability is reduced^(85)^. In this review, food insecure children were more likely to consume unhealthy snacks and fast foods^(31,52,65)^. Unhealthy foods are often cheaper than healthier options, which may contribute to higher obesity rates in food insecure populations who lack the resources to access nutritious food or rely on food banks^(85,86)^. However, food bank items may not be adequately nutritionally balanced, which can negatively affect child diet quality and influence child weight status^(87)^.

Evidence of the association between HFI and fruit and vegetable consumption was mixed^(35,50,72)^, possibly due to caregiver shielding or intra-household HFI, where caregivers may forgo their nutritional needs for their children^(88)^. HFI was associated with reduced family meal participation and meal skipping, possibly due to low availability of food and increased caregiver stress, making preparing family meals more difficult^(24,79,89)^. An evidence gap identified in this review in terms of mechanisms was that no study at the time this review was conducted attempted to investigate the role of diet quality, child mental health or child eating behaviours or parent feeding styles as mediators between HFI and child weight status. A study in UK adults found that HFI was indirectly associated with higher BMI, via distress and eating to cope^(90)^. A similar study approach may be applied to children and adolescents using diet quality, parent feeding styles or child eating behaviours as mediators.

While HFI was associated with untreated dental caries, it was not associated with diet quality, where diet quality was ruled out as a mediator of this relationship^(15)^. Other non-dietary mediators that could explain this relationship may be barriers to accessing oral healthcare products (e.g. toothbrushing)^(91)^. Parental stress experienced during food insecurity may influence caregivers’ ability to encourage dental care. Furthermore, the authors may not have observed an association between diet quality and HFI due to the study’s cross-sectional design, which could not determine causality between HFI, diet and dental health. A more insightful approach would be to analyse longitudinal data on HFI and health outcomes, as this review has established that timing, duration and severity of HFI exposure may have a differential impact on these correlational relationships^(56,82)^.

### Limitations

A limitation of this review was the rapid review study design, which relied on a single reviewer to screen studies. This design may have introduced study selection bias and/or failed to capture all relevant literature and outcomes to fulfil the study aims^(6,92)^. There was a lack of consistency in the measures used to report HFI, with some studies using a validated tool and others using one or two questions within a survey. A recent scoping review commissioned by the Food Standards Agency highlighted the diversity between HFI tools used to measure HFI in the UK, emphasising a need for research groups, governmental departments and third parties investigating food insecurity to report and recognise the strengths and limitations of the methods used and acknowledge discrepancies between different measures^(93)^. Most studies included in this review relied on parent-reported HFI, and incorporating child-reported HFI may provide valuable insights into children’s own experience of HFI, especially in adolescents who may have a greater awareness of HFI and more autonomy over their food environment. Using child-reported measures could offer a more accurate perspective of HFI for developing interventions tailored to the needs of child/adolescent populations^(54,94)^.

It was not appropriate to conduct a meta-analysis due to the heterogeneity between outcomes measured, population selected, study comparators and the varied instruments used to measure HFI across included studies. Additionally, a meta-analysis approach would be overly simplistic to capture the complex systems that impact the relationships between HFI and child/adolescent health outcomes^(95)^. In this study, statistical significance was used as a practical tool to conceptualise HFI and its associated health outcomes. While this approach may be considered controversial in light of emerging literature suggesting that over-reliance on statistical significance can lead to incorrect conclusions, effect sizes were also reported to provide a more nuanced understanding of the correlational relationships between HFI and child/adolescent health^(96)^. This dual approach ensured a balanced interpretation of mechanisms and associations while offering a foundation for further exploration of these complex relationships.

### Implications of this review

The review identified a gap in the literature for UK-based studies and highlighted that further research using quantitative methods and longitudinal data may be beneficial for gaining more insight into the mechanisms by which HFI is associated with the plethora of outcomes identified in this review. Due to the inclusion criteria, qualitative evidence and studies of LMIC were excluded. This review can be used to inform future research priorities, such as a qualitative review, which could add depth and accounts of individual experiences of HFI to supplement the findings of this review. Additionally, a review exploring HFI and child/adolescent outcomes and mediators in LMIC could be conducted to compare findings.

The conceptual map provides a guide for policymakers to identify where interventions may be beneficial in ameliorating the health impact of HFI in children and adolescents in Western HIC. The study scope was limited to children and adolescents in Western HIC, physical and mental health outcomes and biological processes (e.g. sleep) to reduce the impact of heterogeneity and improve the validity of the results. Given the complexity of the problem of HFI, the findings may not be appropriate for supporting interventions and policies in other settings (e.g. LMIC), for younger child age groups (e.g. infants < 3 years) and for health outcomes that are not explicitly summarised in this review. Policymakers should be aware of these limitations when using this review as evidence for intervention development. However, the results from this review can help guide further research in other settings and child populations.

### Conclusions

The present rapid review identified that HFI is related to detrimental child physical and psychosocial health outcomes via (i) diet and (ii) mental health pathways. Maternal mental health and parent stress were identified as mediators explaining the relationship between HFI and child/adolescent behaviour and mental health. A paucity of longitudinal studies and studies of UK child populations highlights evidence gaps and priorities for further research. Sociodemographic factors such as ethnicity and household income were identified as key determinants of HFI, and policymakers should take these into account when planning interventions aiming to improve health in food insecure child populations. Additionally, supplementation of this quantitative review with qualitative evidence will provide a complete picture of this research problem.

## Supporting information

Abraham et al. supplementary material Abraham et al. supplementary material

## Supplementary material

For supplementary material accompanying this paper, visit https://doi.org/10.1017/S1368980025101092.
