# Supplementary material for: Household food insecurity and its impact on child and adolescent health outcomes in Western high-income countries: a rapid review of mechanisms and associations
Source: Public Health Nutr. 2025 Aug 19;29(1):e17. doi: 10.1017/S1368980025101092 (PMC12895477; doi:10.1017/S1368980025101092)
Supplement: Abraham et al. supplementary material [file S1368980025101092sup001.docx]

**Supplementary material**

QUIPS forms for all mechanism studies.

| **Author and year of publication** | **Banahan 2021** | | | |
| --- | --- | --- | --- | --- |
| **Study identifier** | Sarah Abraham | | | |
| **Reviewer** |  |  |  |  |
|  |  |  |  |  |
| **Biases** | **Issues to consider for judging overall rating of "Risk of bias"** | **Study Methods & Comments** | **Rating of reporting** | **Rating of "Risk of bias"** |
| Instructions to assess the risk of each potential bias: | These issues will guide your thinking and judgment about the overall risk of bias within each of the 6 domains. Some 'issues' may not be relevant to the specific study or the review research question. These issues are taken together to inform the overall judgment of potential bias for each of the 6 domains. | Provide comments or text exerpts in the white boxes below, as necessary, to facilitate the consensus process that will follow. | Click on each of the blue cells and choose from the drop down menu to rate the adequacy of reporting as yes, partial, no or unsure. | Click on the green cells; choose from the drop-down menu to rate potential risk of bias for each of the 6 domains as High, Moderate, or Low considering all relevant issues |
| **1. Study Participation** | **Goal: To judge the risk of selection bias (likelihood that relationship between *PF* and**  ***outcome* is different for participants and eligible non-participants).** |  |  |  |
| *Source of target population* | The source population or population of interest is adequately described for key characteristics (LIST). | The population used for the study was sourced from the nationally representative population data from NHANES cycles 2011-2012 and 2013/2014. The key characteristics were children aged 5-7 years old and their parents. Their gender, race/ ethnicity/ household income, household size, SNAP programme participation and health outcomes information was included. | Yes |  |
| *Method used to identify population* | The sampling frame and recruitment are adequately described, including methods to identify the sample  sufficient to limit potential bias (number and type used, e.g., referral patterns in health care) | Each NHANES cycle provides data to assess the health and nutrition of children and adults residing in all 50 states and Washington DC. Selection of participants is based on a complex, multistage, probability sampling design in order to represent the noninstitutionalized US civilian population. | Yes |  |
| *Recruitment period* | Period of recruitment is adequately described | NHANES recruitment 2011/2012 cycles and 2013/2014 cycles | Yes |  |
| *Place of recruitment* | Place of recruitment (setting and geographic location) are adequately described | In home interviews and physical medical assessments (in mobile examination units). Diet was measured by trained interviewers using the NHANES computer-assisted dietary interview system. | Yes |  |
| *Inclusion and exclusion criteria* | Inclusion and exclusion criteria are adequately described (e.g., including explicit diagnostic criteria or “zero time” description). | The study population included children who had completed HFI surveys, dietary surveys and dental examinations and had at least 2 teeth. | Yes |  |
| *Adequate study participation* | There is adequate participation in the study by eligible individuals | Yes, this is a survey dataset (n=4822 children) | Yes |  |
| *Baseline characteristics* | The baseline study sample (i.e., individuals entering the study) is adequately described for key characteristics  (LIST). | 22.4% of the sample were aged 5-7, 30.02% were 8-11 years and 47.56% were 12-17 years. There was even split between males and females. 53% of the sample were White, 14% were Black and 24% were Hispanic, with 9% reporting “Other” ethnicity. 17% of the sample had an annual income <20,000. The mean house size was 4.5 (sd=0.03). 87% of the sample did not receive SNAP. Majority of the sample were fully food secure (57%) | Yes |  |
| **Summary Study participation** | **The study sample represents the population of interest on key characteristics, sufficient to limit**  **potential bias of the observed relationship between PF and outcome.** | Low risk of bias was presented for the study participants. The study included population data from a large nationally representative survey. There was an adequate number of food insecure vs food secure within the population. A low risk of bias between the PF and outcome was concluded. | Yes | Low |
|  | | | | |
| **2. Study Attrition** | **Goal: To judge the risk of attrition bias (likelihood that relationship between *PF* and**  ***outcome* are different for completing and non-completing participants).** |  |  |  |
| *Proportion of baseline sample available*  *for analysis* | Response rate (i.e., proportion of study sample completing the study and providing outcome data) is adequate. | Adequate sample only samples for which data exists are included. Overall response rate of NHANES is around 70-80% | Yes |  |
| *Attempts to collect information on*  *participants who dropped out* | Attempts to collect information on participants who dropped out of the study are described. | This was a secondary analysis of a cross-sectional survey so no loss of drop out is reported. | Yes |  |
| *Reasons and potential impact of*  *subjects lost to follow-up* | Reasons for loss to follow-up are provided. | N/A | Yes |  |
| *Outcome and prognostic factor information on those lost to follow-up* | Participants lost to follow-up are adequately described for key characteristics (LIST). | N/A | Yes |  |
|  | There are no important differences between key characteristics (LIST) and outcomes in participants who  completed the study and those who did not. | N/A | Yes |  |
| **Study Attrition Summary** | **Loss to follow-up (from baseline sample to study population analyzed) is not associated with key characteristics (i.e., the study data adequately represent the sample) sufficient to limit potential bias to the observed relationship between PF and outcome.** | There is a low risk of bias as the study used complete data from the NHANEs survey. This was a cross-sectional study and loss-to follow up was considered non-applicable. | Yes | Low |
|  | | | | |
| **3. Prognostic Factor**  **Measurement** | **Goal: To judge the risk of measurement bias related to how PF was measured (differential measurement of PF related to the level of outcome).** |  |  |  |
| *Definition of the PF* | A clear definition or description of 'PF' is provided (e.g., including dose, level, duration of exposure, and clear  specification of the method of measurement). | Yes, food insecurity is measured by the USDA approved 18 item food insecurity measure for household food insecurity. | Yes |  |
| *Valid and Reliable Measurement of PF* | Method of PF measurement is adequately valid and reliable to limit misclassification bias (e.g., may include relevant outside sources of information on measurement properties, also characteristics, such as blind  measurement and limited reliance on recall). | Yes, it is a valid method of measurement and is parent reported it. The survey asks parents of HFI experienced over the past 12 months. | Yes |  |
|  | Continuous variables are reported or appropriate cut-points (i.e., not data-dependent) are used. | The raw scores obtained from the completed HFI surveys are summed and the sum of the affirmative responses is translated into the severity of HFI.  Cut offs based on affirmative responses – full (no responses), marginal (1-2 responses), low (3-7 responses), or very low (8 or more responses) | Yes |  |
| *Method and Setting of PF Measurement* | The method and setting of measurement of PF is the same for all study participants. | The survey was conducted in several states the measurements used were the same. | Yes |  |
| *Proportion of data on PF available for*  *analysis* | Adequate proportion of the study sample has complete data for PF variable. | Yes | Yes |  |
| *Method used for missing data* | Appropriate methods of imputation are used for missing 'PF' data. | No missing values | Yes |  |
| **PF Measurement Summary** | ***PF* is adequately measured in study participants to sufficiently limit potential bias.** | Yes, PF is measured by a validated tool and parent reported. A low risk of bias was concluded for PF measurement. |  | Low |
|  | | | | |
| **4. Outcome**  **Measurement** | **Goal: To judge the risk of bias related to the measurement of outcome (differential measurement of outcome related to the baseline level of PF).** |  |  |  |
| *Definition of the Outcome* | A clear definition of outcome is provided, including duration of follow-up and level and extent of the outcome  construct. | Yes – the outcome is one or more untreated dental caries | Yes |  |
| *Valid and Reliable Measurement of Outcome* | The method of outcome measurement used is adequately valid and reliable to limit misclassification bias (e.g., may include relevant outside sources of information on measurement properties, also characteristics, such as blind measurement and confirmation of outcome with valid and reliable test). | Untreated dental caries was used as an indicator of the child’s current dental needs (no carious teeth versus 1 or more carious teeth). Diagnosis was made by diagnostic criteria and carried out by licensed examiners. Untreated dental caries was used instead of decayed, missing and filed teeth index to represent a measure of the current dental need rather than past caries. | Unsure |  |
| *Method and Setting of Outcome*  *Measurement* | The method and setting of outcome measurement is the same for all study participants. | The survey was conducted in several states however the measurements used were the same. | Yes |  |
| **Outcome Measurement Summary** | ***Outcome of interest* is adequately measured in study participants to sufficiently limit potential bias.** | Yes, there is sufficient evidence which limits measurement bias of the outcomes of interest in this study. | Yes | Low |
|  | | | | |
| **5. Study Confounding** | **Goal: To judge the risk of bias due to confounding (i.e. the effect of PF is distorted by**  **another factor that is related to PF and outcome).** |  |  |  |
| *Important Confounders Measured* | All important confounders, including treatments (key variables in conceptual model: LIST), are measured. | Yes, confounders such as age; sex, race and ethnicity, household income, household size, snap participation, oral healthcare visits and total number of teeth | Yes |  |
| *Definition of the confounding factor* | Clear definitions of the important confounders measured are provided (e.g., including dose, level, and duration  of exposures). | Yes  Confounders such as age and sex explain themselves.  Supplemental Nutrition Assistance Programme (SNAP) supplement the budget for food for households so they can purchase healthy foods for all members and can be considered a marker of SES. | Yes |  |
| *Valid and Reliable Measurement of Confounders* | Measurement of all important confounders is adequately valid and reliable (e.g., may include relevant outside sources of information on measurement properties, also characteristics, such as blind measurement and limited  reliance on recall). | The NHANES survey is self-reported for sociodemographic characteristics. | Yes |  |
| *Method and Setting of Confounding*  *Measurement* | The method and setting of confounding measurement are the same for all study participants. | The survey was conducted in several states however the measurements used were the same. | Unsure |  |
| *Method used for missing data* | Appropriate methods are used if imputation is used for missing confounder data. | N/A | Yes |  |
| *Appropriate Accounting for Confounding* | Important potential confounders are accounted for in the study design (e.g., matching for key variables,  stratification, or initial assembly of comparable groups). | Yes all of the key confounders as mentioned above. | Yes |  |
|  | Important potential confounders are accounted for in the analysis (i.e., appropriate adjustment). | Yes | Yes |  |
| **Study Confounding Summary** | **Important potential confounders are appropriately accounted for, limiting potential bias with respect to**  **the relationship between *PF* and *outcome* .** | Due to the study using NHANES data which is a routine survey conducted by train staff and validated surveys, there is low risk of bias for the measurement of confounders. All of the appropriate confounders are included to adjust for the relationship between HFI and the primary outcome. | Yes | Low |
|  | | | | |
| **6. Statistical Analysis**  **and Reporting** | **Goal: To judge the risk of bias related to the statistical analysis and presentation of results.** |  |  |  |
| *Presentation of analytical strategy* | There is sufficient presentation of data to assess the adequacy of the analysis. | Yes sufficient data was presented (n=4822) | Yes |  |
| *Model development strategy* | The strategy for model building (i.e., inclusion of variables in the statistical model) is appropriate and is based  on a conceptual framework or model. | Yes the model is mediation model based on the literature that suggests HFI is associated with dental caries via poor diet quality. | Yes |  |
|  | The selected statistical model is adequate for the design of the study. | Yes a primary mediation model was selected. However the study uses the Baron and Kenny method which is considered an outdated approach. | Yes |  |
| *Reporting of results* | There is no selective reporting of results. | No | Yes |  |
| **Statistical Analysis and Presentation**  **Summary** | **The statistical analysis is appropriate for the design of the study, limiting potential for presentation of**  **invalid or spurious results.** | The analysis was appropriate for the design of the study, however the mediation model used was outdated therefore a moderate bias score is concluded. |  | Moderate |

| **Author and year of publication** | **Do et al., 2021 - LOW BIAS** | | | | |  |
| --- | --- | --- | --- | --- | --- | --- |
| **Study identifier** | Sarah Abraham | | | | |  |
| **Reviewer** |  |  |  |  |  |  |
|  |  | |  |  |  |  |
| **Biases** | **Issues to consider for judging overall rating of "Risk of bias"** | | **Study Methods & Comments** | **Rating of reporting** | **Rating of "Risk of bias"** |  |
| Instructions to assess the risk of each potential bias: | These issues will guide your thinking and judgment about the overall risk of bias within each of the 6 domains. Some 'issues' may not be relevant to the specific study or the review research question. These issues are taken together to inform the overall judgment of potential bias for each of the 6 domains. | | Provide comments or text exerpts in the white boxes below, as necessary, to facilitate the consensus process that will follow. | Click on each of the blue cells and choose from the drop down menu to rate the adequacy of reporting as yes, partial, no or unsure. | Click on the green cells; choose from the drop-down menu to rate potential risk of bias for each of the 6 domains as High, Moderate, or Low considering all relevant issues |  |
| **1. Study Participation** | **Goal: To judge the risk of selection bias (likelihood that relationship between *PF* and**  ***outcome* is different for participants and eligible non-participants).** | |  |  |  |  |
| *Source of target population* | The source population or population of interest is adequately described for key characteristics (LIST). | | Yes the information provided is: information is obtained from the FLASHE study. FLASHE contains child-parent dyads of children aged 12-17 years old. Information obtained: sleep measures, weight status, sex, race/ethnicity, parents weight status, parents’ education, parents income level and household food insecurity levels. | Yes |  |  |
| *Method used to identify population* | The sampling frame and recruitment are adequately described, including methods to identify the sample  sufficient to limit potential bias (number and type used, e.g., referral patterns in health care) | | Yes – the FLASHE study N=1945 dyads of 5027 dyads were enrolled. N=1890 of the N=1945 had complete survey data. N=1544 adolescents who had BMI and sleep measures were included in the analytic sample.  Sample for the study was drawn from the consumer opinion panel representative of the US population for sex, education, income, household size and region.  Receiving the diet survey was through DYAD randomization. | Yes |  |  |
| *Recruitment period* | Period of recruitment is adequately described | | April- October 2014 | Yes |  |  |
| *Place of recruitment* | Place of recruitment (setting and geographic location) are adequately described | | This was a cross-sectional study that was web-based. Invitations were sent to parents and adolescents email addresses. | Yes |  |  |
| *Inclusion and exclusion criteria* | Inclusion and exclusion criteria are adequately described (e.g., including explicit diagnostic criteria or “zero time” description). | | Parent-child dyads were recruited if the parent was over 18 and lived with the adolescent 50% or greater of the time. A third of adolescent participants were recruited to be part of three age ranges and evenly split by gender. Only participants with complete BMI and sleep data were included in the analytic sample (n=1544). | Yes |  |  |
| *Adequate study participation* | There is adequate participation in the study by eligible individuals | | Yes, this is a survey dataset | Yes |  |  |
| *Baseline characteristics* | The baseline study sample (i.e., individuals entering the study) is adequately described for key characteristics  (LIST). | | In the sample 64% of the population were White, 9.9% Hispanic, 16.2% were Black and 9.3% Other. The mean age of adolescents was 14.5 (SD=1.6). Majority of households had parents with four-year college degree or higher (47.1%) and only 7% had less than a college degree. Income was based on two scales (household earnings of $0-99,999 and $100,000+) where 79% of households were in the $0-99,999 category. | Yes |  |  |
| **Summary Study participation** | **The study sample represents the population of interest on key characteristics, sufficient to limit**  **potential bias of the observed relationship between PF and outcome.** | | Low risk of bias was concluded for the study participants (and non-participants). |  | Low |  |
|  | | | | | |  |
| **2. Study Attrition** | **Goal: To judge the risk of attrition bias (likelihood that relationship between *PF* and**  ***outcome* are different for completing and non-completing participants).** | |  |  |  |  |
| *Proportion of baseline sample available*  *for analysis* | Response rate (i.e., proportion of study sample completing the study and providing outcome data) is adequate. | | N=1945 dyads of 5027 dyads were enrolled. N=1890 of the N=1945 had complete survey data. N=1544 adolescents who had BMI and sleep measures were included in the analytic sample. | Yes |  |  |
| *Attempts to collect information on*  *participants who dropped out* | Attempts to collect information on participants who dropped out of the study are described. | | After closure of the enrollment period no more participants were enrolled, they were only enrolled once in this study. In the initial enrollment period, follow up emails letters and phone messages were sent to potential participants if they had not responded. Enrollment period started in April 2014-October 2014. | N/A yes |  |  |
| *Reasons and potential impact of*  *subjects lost to follow-up* | Reasons for loss to follow-up are provided. | | N/A | N/A yes |  |  |
| *Outcome and prognostic factor information on those lost to follow-up* | Participants lost to follow-up are adequately described for key characteristics (LIST). | | N/A | N/A yes |  |  |
|  | There are no important differences between key characteristics (LIST) and outcomes in participants who  completed the study and those who did not. | | N/A |  |  |  |
| **Study Attrition Summary** | **Loss to follow-up (from baseline sample to study population analyzed) is not associated with key characteristics (i.e., the study data adequately represent the sample) sufficient to limit potential bias to the observed relationship between PF and outcome.** | | Loss to follow not applicable for this dataset as this was a secondary analysis of cross-sectional data. |  | Low |  |
|  | | | | | |  |
| **3. Prognostic Factor**  **Measurement** | | **Goal: To judge the risk of measurement bias related to how PF was measured (differential measurement of PF related to the level of outcome).** |  |  |  | |
| ***Definition of the PF*** | | **A clear definition or description of 'PF' is provided (e.g., including dose, level, duration of exposure, and clear**  **specification of the method of measurement).** | Yes, it is food insecurity, and the method of measurement is provided – a 2-item validated screener survey was used to measure HFI. The method had a sensitivity of 97% and specificity of 83% in determining food insecurity. | **Yes** |  | |
| ***Valid and Reliable Measurement of PF*** | | **Method of PF measurement is adequately valid and reliable to limit misclassification bias (e.g., may include relevant outside sources of information on measurement properties, also characteristics, such as blind**  **measurement and limited reliance on recall).** | Yes, it is a valid method of measurement which was parent completed asking two questions:   1. Within the past 12 months we worried whether our food would run out before we got money to buy more” 2. Within the past 12 months, the food we bought just didn’t last and we didn’t have money to get more.   The parents could respond with three statements: never true, sometimes true and often true. | **Yes** |  | |
|  |  | **Continuous variables are reported or appropriate cut-points (i.e., not data-dependent) are used.** | Each of the items were dichotomized for never (0) and sometimes/often true (1). Answering 1 to either question was indicative of food security, whereas 0 was indicative of food security. | **Yes** |  | |
| ***Method and Setting of PF Measurement*** | | **The method and setting of measurement of PF is the same for all study participants.** | Yes | **Yes** |  | |
| ***Proportion of data on PF available for***  ***analysis*** | | **Adequate proportion of the study sample has complete data for PF variable.** | Yes | **Yes** |  | |
| ***Method used for missing data*** | | **Appropriate methods of imputation are used for missing 'PF' data.** | N/A | **Yes** |  | |
| **PF Measurement Summary** | | ***PF* is adequately measured in study participants to sufficiently limit potential bias.** | Yes the PF measure was adequately measured by a validated measure for the sample. |  | **Low** | |
|  | | | | | | |
| **4. Outcome**  **Measurement** | | **Goal: To judge the risk of bias related to the measurement of outcome (differential measurement of outcome related to the baseline level of PF).** |  |  |  | |
| ***Definition of the Outcome*** | | **A clear definition of outcome is provided, including duration of follow-up and level and extent of the outcome**  **construct.** | Weight status was examined using BMI and categorised according to age and gender specific charts by the CDC.  Sleep was examined using two questions which ask whether the child has regular bedtimes, whether they have difficulty staying asleep and calculating average nighttime sleep duration. | **Yes** |  | |
| ***Valid and Reliable Measurement of Outcome*** | | **The method of outcome measurement used is adequately valid and reliable to limit misclassification bias (e.g., may include relevant outside sources of information on measurement properties, also characteristics, such as blind measurement and confirmation of outcome with valid and reliable test).** | Weight status – was categorised using the adolescent BMI using CDC growth charts based on age and sex.  Sleep was examined using two questions which ask whether the child has regular bedtimes, whether they have difficulty staying asleep and calculating average nighttime sleep duration. | **No to sleep questions** |  | |
| ***Method and Setting of Outcome***  ***Measurement*** | | **The method and setting of outcome measurement is the same for all study participants.** | Yes the data was obtained from the | **Yes** |  | |
| **Outcome Measurement Summary** | | ***Outcome of interest* is adequately measured in study participants to sufficiently limit potential bias.** | **Yes, the outcomes of interest were adequately measured concluding low bias** |  | **Low** | |
|  | | | | | | |
| **5. Study Confounding** | | **Goal: To judge the risk of bias due to confounding (i.e. the effect of PF is distorted by**  **another factor that is related to PF and outcome).** |  |  |  | |
| ***Important Confounders Measured*** | | **All important confounders, including treatments (key variables in conceptual model: LIST), are measured.** | Yes. Age, household income. Race/ethnicity, parent education and income, parent wight status | **Yes** |  | |
| ***Definition of the confounding factor*** | | **Clear definitions of the important confounders measured are provided (e.g., including dose, level, and duration**  **of exposures).** | Yes, this has been provided.  Household income was placed into categories, Race/ethnicity was categorised into White, Black, Hispanic and Other. Parent education had several categories from less than high school education to 4y ear + college degree. | **Yes** |  | |
| ***Valid and Reliable Measurement of Confounders*** | | **Measurement of all important confounders is adequately valid and reliable (e.g., may include relevant outside sources of information on measurement properties, also characteristics, such as blind measurement and limited**  **reliance on recall).** | Yes – self reported using the online questionnaire- race divided into races, parent weight status into categories, and parental education categories. | **Yes** |  | |
| ***Method and Setting of Confounding***  ***Measurement*** | | **The method and setting of confounding measurement are the same for all study participants.** | Yes the measurements were collected as part of the FLASE study and method of collection was online. | **Not sure** |  | |
| ***Method used for missing data*** | | **Appropriate methods are used if imputation is used for missing confounder data.** | N/A | **N/A** |  | |
| ***Appropriate Accounting for Confounding*** | | **Important potential confounders are accounted for in the study design (e.g., matching for key variables,**  **stratification, or initial assembly of comparable groups).** | Yes | **Yes** |  | |
|  |  | **Important potential confounders are accounted for in the analysis (i.e., appropriate adjustment).** | Yes | **Yes** |  | |
| **Study Confounding Summary** | | **Important potential confounders are appropriately accounted for, limiting potential bias with respect to**  **the relationship between *PF* and *outcome* .** | Yes, important confounders are accounted for low bias is concluded. |  | **Low** | |
|  | | | | | | |
| **6. Statistical Analysis**  **and Reporting** | | **Goal: To judge the risk of bias related to the statistical analysis and presentation of results.** |  |  |  | |
| ***Presentation of analytical strategy*** | | **There is sufficient presentation of data to assess the adequacy of the analysis.** | Yes the analytic sample was n=1544. | **Yes** |  | |
| ***Model development strategy*** | | **The strategy for model building (i.e., inclusion of variables in the statistical model) is appropriate and is based**  **on a conceptual framework or model.** | Conditional processes models were used to investigate associations between sleep measure and weight status and food insecurity as a mediator between this relationship is also investigated along with sex.  Conditional processes models were used to investigate association between food insecurity and weight status and sleep was investigated as mediator between this association.  Models controlled for the effects of age, race/ethnicity, parent education, income, and weight status.  Significance tested as the p-value of 0.05. | **Yes** |  | |
|  |  | **The selected statistical model is adequate for the design of the study.** | **Yes** | **Yes** |  | |
| ***Reporting of results*** | | **There is no selective reporting of results.** | No all results were made transparent in the study results section. | **Yes** |  | |
| **Statistical Analysis and Presentation**  **Summary** | | **The statistical analysis is appropriate for the design of the study, limiting potential for presentation of**  **invalid or spurious results.** | Yes, the statistical analysis was considered appropriate for the design of the study and limited the potential presentation of invalid results. Low bias was concluded. |  | **Low** | |

| **Author and year of publication** | **Gee and Asim (2019)** | | | | |
| --- | --- | --- | --- | --- | --- |
| **Study identifier** | Sarah Abraham | | | | |
| **Reviewer** |  |  |  |  |  |
|  |  | |  |  |  |
| **Biases** | **Issues to consider for judging overall rating of "Risk of bias"** | | **Study Methods & Comments** | **Rating of reporting** | **Rating of "Risk of bias"** |
| Instructions to assess the risk of each potential bias: | These issues will guide your thinking and judgment about the overall risk of bias within each of the 6 domains. Some 'issues' may not be relevant to the specific study or the review research question. These issues are taken together to inform the overall judgment of potential bias for each of the 6 domains. | | Provide comments or text exerpts in the white boxes below, as necessary, to facilitate the consensus process that will follow. | Click on each of the blue cells and choose from the drop down menu to rate the adequacy of reporting as yes, partial, no or unsure. | Click on the green cells; choose from the drop-down menu to rate potential risk of bias for each of the 6 domains as High, Moderate, or Low considering all relevant issues |
| **1. Study Participation** | **Goal: To judge the risk of selection bias (likelihood that relationship between *PF* and**  ***outcome* is different for participants and eligible non-participants).** | |  |  |  |
| *Source of target population* | The source population or population of interest is adequately described for key characteristics (LIST). | | Yes, the source population is adequately described. Children from across the US who entered kindergarten in fall 2010 are included. Children and their parents are included in the study. The data contained a robust set of measures for HFI, parenting and behaviour as well as key sociodemographic characteristics. | Yes |  |
| *Method used to identify population* | The sampling frame and recruitment are adequately described, including methods to identify the sample  sufficient to limit potential bias (number and type used, e.g., referral patterns in health care) | | Yes – longitudinal data from the Early Childhood Longitudinal Study Kindergarten class of 2010-2011was used for the analysis. | Yes |  |
| *Recruitment period* | Period of recruitment is adequately described | | Yes 2010-2011 | Yes |  |
| *Place of recruitment* | Place of recruitment (setting and geographic location) are adequately described | | Yes USA nationally representative data set collected from kindergarten and schools | Yes |  |
| *Inclusion and exclusion criteria* | Inclusion and exclusion criteria are adequately described (e.g., including explicit diagnostic criteria or “zero time” description). | | A sample of 7,820 of children and adults were selected by excluding those in the main sample of 18,200 who had a zero sampling weight (so any child with missing survey component had a zero weight and were not part of the analytic survey) | Yes |  |
| *Adequate study participation* | There is adequate participation in the study by eligible individuals | | Yes this is a survey dataset | Yes |  |
| *Baseline characteristics* | The baseline study sample (i.e., individuals entering the study) is adequately described for key characteristics  (LIST). | | Yes, information was provided for household characteristics including socioeconomic status, access to medical care, employment, receipt of food stamps, ethnicity, and how involved the parents were in the school. | Yes |  |
| **Summary Study participation** | **The study sample represents the population of interest on key characteristics, sufficient to limit**  **potential bias of the observed relationship between PF and outcome.** | | The study sample adequately represented US infants and their parents and the sample was nationally representative. Low bias was concluded for the study. |  | Low |
|  | | | | | |
| **2. Study Attrition** | **Goal: To judge the risk of attrition bias (likelihood that relationship between *PF* and**  ***outcome* are different for completing and non-completing participants).** | |  |  |  |
| *Proportion of baseline sample available*  *for analysis* | Response rate (i.e., proportion of study sample completing the study and providing outcome data) is adequate. | | 7,820 out of 18,200 had completed results. Missing data were still present among this sample ranging from 0 to 13%. 30% of children had missing data on one or more variable | Yes |  |
| *Attempts to collect information on*  *participants who dropped out* | Attempts to collect information on participants who dropped out of the study are described. | | No this is not described | No |  |
| *Reasons and potential impact of*  *subjects lost to follow-up* | Reasons for loss to follow-up are provided. | | No this is not described | No |  |
| *Outcome and prognostic factor information on those lost to follow-up* | Participants lost to follow-up are adequately described for key characteristics (LIST). | | No this is not described | No |  |
|  | There are no important differences between key characteristics (LIST) and outcomes in participants who  completed the study and those who did not. | | Not provided | Unsure |  |
| **Study Attrition Summary** | **Loss to follow-up (from baseline sample to study population analyzed) is not associated with key characteristics (i.e., the study data adequately represent the sample) sufficient to limit potential bias to the observed relationship between PF and outcome.** | | High risk of bias was concluded as no information was provided for the missing data in the study sample. |  | High |
|  | | | | | |
| **3. Prognostic Factor**  **Measurement** | | **Goal: To judge the risk of measurement bias related to how PF was measured (differential measurement of PF related to the level of outcome).** |  |  |  |
| ***Definition of the PF*** | | **A clear definition or description of 'PF' is provided (e.g., including dose, level, duration of exposure, and clear**  **specification of the method of measurement).** | Yes the definition was adequately provided as “In the United States, approximately 6.5 million children were from homes where both children and adults in the home experienced *food insecurity* (U.S. Department of Agriculture, 2018) a phenomenon whereby families face uncertainty in obtaining food due to lack of resources, including the monetary means to acquire nutritionally acceptable and safe foods” | **Yes** |  |
| ***Valid and Reliable Measurement of PF*** | | **Method of PF measurement is adequately valid and reliable to limit misclassification bias (e.g., may include relevant outside sources of information on measurement properties, also characteristics, such as blind**  **measurement and limited reliance on recall).** | Yes, a validated measure of HFI was used– it was administered to parents twice, once when their child was in the spring of kindergarten and a year later when their child was in spring of first grade | **Yes** |  |
|  |  | **Continuous variables are reported or appropriate cut-points (i.e., not data-dependent) are used.** | Dichotomous variable of HFI was created using raw scores from the HFSSM based on cut-offs established by the USDA – raw score 0 to 2 (food secure) raw score 3-10 (food insecure) | **Yes** |  |
| ***Method and Setting of PF Measurement*** | | **The method and setting of measurement of PF is the same for all study participants.** | Yes, kindergarten and school setting for teachers and parents assessed via telephone call | **Yes** |  |
| ***Proportion of data on PF available for***  ***analysis*** | | **Adequate proportion of the study sample has complete data for PF variable.** | This is unclear | **Unsure** |  |
| ***Method used for missing data*** | | **Appropriate methods of imputation are used for missing 'PF' data.** | Yes - multiple imputation by chained equations was used and generated 30 imputed data sets as 30% of the children in the data had missing data on or one or more variables | **Yes** |  |
| **PF Measurement Summary** | | ***PF* is adequately measured in study participants to sufficiently limit potential bias.** | **HFI was adequately measured however it was unclear how much of the study had missing HFI data therefore moderate bias has been concluded for this.** |  | **Moderate** |
|  | | | | | |
| **4. Outcome**  **Measurement** | | **Goal: To judge the risk of bias related to the measurement of outcome (differential measurement of outcome related to the baseline level of PF).** |  |  |  |
| ***Definition of the Outcome*** | | **A clear definition of outcome is provided, including duration of follow-up and level and extent of the outcome**  **construct.** | Yes – the outcome is parenting aggravation and children’s behavior observed over time from kindergarten and spring first grade with food security being used as the main predictor.  Adequate description was provided for internalising and externalising scores. | **Yes** |  |
| ***Valid and Reliable Measurement of Outcome*** | | **The method of outcome measurement used is adequately valid and reliable to limit misclassification bias (e.g., may include relevant outside sources of information on measurement properties, also characteristics, such as blind measurement and confirmation of outcome with valid and reliable test).** | Aggravation in Parenting Scale – originally derived from the parenting stress index have been used in several national level surveys including National Survey of Americas Families and the US Department of Health and Human Services National Survey of Children’s Health. Method used here was consistent with previous methods used in this research area.  Child behaviour was teacher reported, and parent reported based on Social Skills Rating system | **Yes** |  |
| ***Method and Setting of Outcome***  ***Measurement*** | | **The method and setting of outcome measurement is the same for all study participants.** | **Yes** | **Yes** |  |
| **Outcome Measurement Summary** | | ***Outcome of interest* is adequately measured in study participants to sufficiently limit potential bias.** | **Yes** |  | **Low** |
|  | | | | | |
| **5. Study Confounding** | | **Goal: To judge the risk of bias due to confounding (i.e. the effect of PF is distorted by**  **another factor that is related to PF and outcome).** |  |  |  |
| ***Important Confounders Measured*** | | **All important confounders, including treatments (key variables in conceptual model: LIST), are measured.** | The confounders include ethnicity, parent marital status, employment, food stamps, number of siblings in a child’s household and the household’s socioeconomic status | **Yes** |  |
| ***Definition of the confounding factor*** | | **Clear definitions of the important confounders measured are provided (e.g., including dose, level, and duration**  **of exposures).** | Yes, these are provided in the supplementary material. | **Yes** |  |
| ***Valid and Reliable Measurement of Confounders*** | | **Measurement of all important confounders is adequately valid and reliable (e.g., may include relevant outside sources of information on measurement properties, also characteristics, such as blind measurement and limited**  **reliance on recall).** | Yes, socioeconomic status was measure by the National Center for Educational statistics continuous index which consisted of a composite of parent’s education level, occupational prestige and income. Access to medical care was also included and based on whether the child had health insurance. | **Yes** |  |
| ***Method and Setting of Confounding***  ***Measurement*** | | **The method and setting of confounding measurement are the same for all study participants.** | This is unclear. | **Unclear** |  |
| ***Method used for missing data*** | | **Appropriate methods are used if imputation is used for missing confounder data.** | Yes multiple imputation by chained equations was used to account for missing data. | **Yes** |  |
| ***Appropriate Accounting for Confounding*** | | **Important potential confounders are accounted for in the study design (e.g., matching for key variables,**  **stratification, or initial assembly of comparable groups).** | N/a secondary analysis of data | **Yes** |  |
|  |  | **Important potential confounders are accounted for in the analysis (i.e., appropriate adjustment).** | Yes they were used to adjust the SEM | **Yes** |  |
| **Study Confounding Summary** | | **Important potential confounders are appropriately accounted for, limiting potential bias with respect to**  **the relationship between *PF* and *outcome* .** | Yes, low bias concluded. |  | **Low** |
|  | | | | | |
| **6. Statistical Analysis**  **and Reporting** | | **Goal: To judge the risk of bias related to the statistical analysis and presentation of results.** |  |  |  |
| ***Presentation of analytical strategy*** | | **There is sufficient presentation of data to assess the adequacy of the analysis.** | Yes data on all important variables was presented and the model design was explained. | **Yes** |  |
| ***Model development strategy*** | | **The strategy for model building (i.e., inclusion of variables in the statistical model) is appropriate and is based**  **on a conceptual framework or model.** | The strategy for the model building was based on the Family Stress model which is a well-known theoretical framework. | **Yes** |  |
|  |  | **The selected statistical model is adequate for the design of the study.** | Yes mediation model was used | **Yes** |  |
| ***Reporting of results*** | | **There is no selective reporting of results.** | **No** | **Yes** |  |
| **Statistical Analysis and Presentation**  **Summary** | | **The statistical analysis is appropriate for the design of the study, limiting potential for presentation of**  **invalid or spurious results.** | **Low bias was concluded for the stats analysis and presentation summary.** |  | **Low** |

| **Author and year of publication** | **Gundersen 2008a.** | | | |
| --- | --- | --- | --- | --- |
| **Study identifier** | Sarah Abraham | | | |
| **Reviewer** |  |  |  |  |
|  |  |  |  |  |
| **Biases** | **Issues to consider for judging overall rating of "Risk of bias"** | **Study Methods & Comments** | **Rating of reporting** | **Rating of "Risk of bias"** |
| Instructions to assess the risk of each potential bias: | These issues will guide your thinking and judgment about the overall risk of bias within each of the 6 domains. Some 'issues' may not be relevant to the specific study or the review research question. These issues are taken together to inform the overall judgment of potential bias for each of the 6 domains. | Provide comments or text exerpts in the white boxes below, as necessary, to facilitate the consensus process that will follow. | Click on each of the blue cells and choose from the drop down menu to rate the adequacy of reporting as yes, partial, no or unsure. | Click on the green cells; choose from the drop-down menu to rate potential risk of bias for each of the 6 domains as High, Moderate, or Low considering all relevant issues |
| **1. Study Participation** | **Goal: To judge the risk of selection bias (likelihood that relationship between *PF* and**  ***outcome* is different for participants and eligible non-participants).** |  |  |  |
| *Source of target population* | The source population or population of interest is adequately described for key characteristics (LIST). | Yes population characteristics are described including child obesity status, FI status, maternal stressors, child disability, financial status (working or non working), health insurance, marital status of mother, ratio to poverty line, age of child, child gender, race/ethnicity, BMI of mother, age of mother, education of mother. The source of the population is the NHANES dataset from 1999-2002. | Yes |  |
| *Method used to identify population* | The sampling frame and recruitment are adequately described, including methods to identify the sample  sufficient to limit potential bias (number and type used, e.g., referral patterns in health care) | Each NHANES cycle provides data to assess the health and nutrition of children and adults residing in all 50 states and Washington DC. Selection of participants is based on a complex, multistage, probability sampling design in order to represent the noninstitutionalized US civilian population. | Yes |  |
| *Recruitment period* | Period of recruitment is adequately described | 1999-2002 recruitment cycle | Yes |  |
| *Place of recruitment* | Place of recruitment (setting and geographic location) are adequately described | Yes USA nationally representative data set interviewed at home and physically examined by trained technicians | Yes |  |
| *Inclusion and exclusion criteria* | Inclusion and exclusion criteria are adequately described (e.g., including explicit diagnostic criteria or “zero time” description). | A sample of 841 children aged 3-17 and their mothers with income below 200% of the poverty line were included in the study. | Yes |  |
| *Adequate study participation* | There is adequate participation in the study by eligible individuals | Yes this is a survey dataset and the sample size is reliable for analysis. | Yes |  |
| *Baseline characteristics* | The baseline study sample (i.e., individuals entering the study) is adequately described for key characteristics  (LIST). | Yes, these are the same as the description above | Yes |  |
| **Summary Study participation** | **The study sample represents the population of interest on key characteristics, sufficient to limit**  **potential bias of the observed relationship between PF and outcome.** | Adequate study participation, and low potential of bias will be observed between PF and outcome due to the participating sample. Although there may be some risk (due to the inclusion criteria) that a larger than average HFI population size is achieved but this was one of the aims of the study. Therefore overall low bias is concluded. |  | Low |
|  | | | | |
| **2. Study Attrition** | **Goal: To judge the risk of attrition bias (likelihood that relationship between *PF* and**  ***outcome* are different for completing and non-completing participants).** |  |  |  |
| *Proportion of baseline sample available*  *for analysis* | Response rate (i.e., proportion of study sample completing the study and providing outcome data) is adequate. | N/A the sample was already decided and this is a secondary analysis of cross-sectional data. | Yes |  |
| *Attempts to collect information on*  *participants who dropped out* | Attempts to collect information on participants who dropped out of the study are described. | N/A | N/A yes |  |
| *Reasons and potential impact of*  *subjects lost to follow-up* | Reasons for loss to follow-up are provided. | N/A | N/A yes |  |
| *Outcome and prognostic factor information on those lost to follow-up* | Participants lost to follow-up are adequately described for key characteristics (LIST). | N/A | N/A yes |  |
|  | There are no important differences between key characteristics (LIST) and outcomes in participants who  completed the study and those who did not. | N/A |  |  |
| **Study Attrition Summary** | **Loss to follow-up (from baseline sample to study population analyzed) is not associated with key characteristics (i.e., the study data adequately represent the sample) sufficient to limit potential bias to the observed relationship between PF and outcome.** | Loss to follow not applicable for this dataset as this is a sample taken from cross-sectional data. Low bias concluded for study attrition. |  | Low |
| \| **3. Prognostic Factor**  **Measurement** \| **Goal: To judge the risk of measurement bias related to how PF was measured (differential measurement of PF related to the level of outcome).** \|  \|  \|  \| \| --- \| --- \| --- \| --- \| --- \| \| *Definition of the PF* \| A clear definition or description of 'PF' is provided (e.g., including dose, level, duration of exposure, and clear  specification of the method of measurement). \| First definition: food security was defined at the household level  Second definition: food insecurity was defined with respect to the children in the household, not just the child for whom overweight was considered \| Yes \|  \| \| *Valid and Reliable Measurement of PF* \| Method of PF measurement is adequately valid and reliable to limit misclassification bias (e.g., may include relevant outside sources of information on measurement properties, also characteristics, such as blind  measurement and limited reliance on recall). \| Yes, it is a valid method of measurement by the USDA HFI 18-item survey module, which was parent reported. \| Yes \|  \| \| Continuous variables are reported or appropriate cut-points (i.e., not data-dependent) are used. \| Households with 2 or more affirmative responses was categorized as food secure and a household with 3 or more affirmative responses was categorized as food insecure  Children defined as being food insecure were those for whom 2 or more of the 8 child-specific questions were answered affirmatively. \| Yes \|  \| \| *Method and Setting of PF Measurement* \| The method and setting of measurement of PF is the same for all study participants. \| Yes the same method was used \| Yes \|  \| \| *Proportion of data on PF available for*  *analysis* \| Adequate proportion of the study sample has complete data for PF variable. \| Yes all participants had complete data \| Yes \|  \| \| *Method used for missing data* \| Appropriate methods of imputation are used for missing 'PF' data. \| N/A \| Yes \|  \| \| **PF Measurement Summary** \| ***PF* is adequately measured in study participants to sufficiently limit potential bias.** \| Yes, HFI was adequately measured. Low bias concluded. \|  \| Low \| \|  \| \| \| \| \| \| **4. Outcome**  **Measurement** \| **Goal: To judge the risk of bias related to the measurement of outcome (differential measurement of outcome related to the baseline level of PF).** \|  \|  \|  \| \| *Definition of the Outcome* \| A clear definition of outcome is provided, including duration of follow-up and level and extent of the outcome  construct. \| Weight status was examined using BMI and categorized according to age and gender specific white charts by the CDC  Maternal stressors index was defined by depressed mood questions from the Organization composite international diagnostic interview  Cumulative physical stressors were defined as physical, mental and emotional stressors that impact daily life of the mother and work they can do  Cumulative financial stressor index was measured related to working, inability to work or having a disability  Cumulative family structure stressor index: defined by marital status (more financial, mental and physical stress) due to being a single parent compared to married mothers, however, does not consider other stressors defined by the authors as “stigma, childcare issues and lack of social support” \| Yes \|  \| \| *Valid and Reliable Measurement of Outcome* \| The method of outcome measurement used is adequately valid and reliable to limit misclassification bias (e.g., may include relevant outside sources of information on measurement properties, also characteristics, such as blind measurement and confirmation of outcome with valid and reliable test). \| Cumulative mental stressors index: measured using items from the Organization Composite International Diagnostic Interview  Cumulative physical stressors: measured using three measures (not sure if these are validated) limitations due to health and inability to perform at least 1 activity of normal daily life  Cumulative financial stressor index: three measures, whether mother is employed, out of the labour force, and how many hours she works; whether she is unable to work due to a disability or if she had no health insurance  Cumulative family structure stressor: marital status was used to measure this. \| Yes \|  \| \| *Method and Setting of Outcome*  *Measurement* \| The method and setting of outcome measurement is the same for all study participants. \| Yes \| Yes \|  \| \| **Outcome Measurement Summary** \| ***Outcome of interest* is adequately measured in study participants to sufficiently limit potential bias.** \| Low bias was detected for the outcome measures. \|  \| Low \| \|  \| \| \| \| \| \| **5. Study Confounding** \| **Goal: To judge the risk of bias due to confounding (i.e. the effect of PF is distorted by**  **another factor that is related to PF and outcome).** \|  \|  \|  \| \| *Important Confounders Measured* \| All important confounders, including treatments (key variables in conceptual model: LIST), are measured. \| Yes. Age, household income. Race/ethnicity, maternal specific covariates (high school degree/ education/ BMI and age). \| Yes \|  \| \| *Definition of the confounding factor* \| Clear definitions of the important confounders measured are provided (e.g., including dose, level, and duration  of exposures). \| Yes each was included with appropriate definition. Self-explanatory so will not be written here. \| Yes \|  \| \| *Valid and Reliable Measurement of Confounders* \| Measurement of all important confounders is adequately valid and reliable (e.g., may include relevant outside sources of information on measurement properties, also characteristics, such as blind measurement and limited  reliance on recall). \| Yes – self reported in the NHANES questionnaire and BMI measured by a trained technician \| Yes \|  \| \| *Method and Setting of Confounding*  *Measurement* \| The method and setting of confounding measurement are the same for all study participants. \| Yes in their homes by trained technicians and surveys were self-reported. \| Not sure \|  \| \| *Method used for missing data* \| Appropriate methods are used if imputation is used for missing confounder data. \| N/A \| N/A \|  \| \| *Appropriate Accounting for Confounding* \| Important potential confounders are accounted for in the study design (e.g., matching for key variables,  stratification, or initial assembly of comparable groups). \| Yes \| Yes \|  \| \| Important potential confounders are accounted for in the analysis (i.e., appropriate adjustment). \| Yes the analysis adjusted for all these confounders \| Yes \|  \| \| **Study Confounding Summary** \| **Important potential confounders are appropriately accounted for, limiting potential bias with respect to**  **the relationship between *PF* and *outcome* .** \| Low risk of bias concluded for confounders of HFI and outcome. \|  \| Low \| \|  \| \| \| \| \| \| **6. Statistical Analysis**  **and Reporting** \| **Goal: To judge the risk of bias related to the statistical analysis and presentation of results.** \|  \|  \|  \| \| *Presentation of analytical strategy* \| There is sufficient presentation of data to assess the adequacy of the analysis. \| Yes the study shows descriptive statistics and also results of the primary analysis \| Yes \|  \| \| *Model development strategy* \| The strategy for model building (i.e., inclusion of variables in the statistical model) is appropriate and is based  on a conceptual framework or model. \| Yes 5 models: model 1: food insecurity, model 2: food insecurity and the 4 maternal stressors; model 3: food insecurity, the 4 maternal stressors, interaction of these indices with food insecurity; model 4: food insecurity and a total cumulative maternal stressor index and model 5; food insecurity, the total cumulative maternal stressor index and interaction of this index with food insecurity. The models were informed prior evidence from the literature. \| Yes \|  \| \| The selected statistical model is adequate for the design of the study. \| Yes \| Yes \|  \| \| *Reporting of results* \| There is no selective reporting of results. \| No selective reporting was detected. \| Yes \|  \| \| **Statistical Analysis and Presentation**  **Summary** \| **The statistical analysis is appropriate for the design of the study, limiting potential for presentation of**  **invalid or spurious results.** \| Low risk of bias was concluded. \|  \| Low \| | | | | |

| **Author and year of publication** | **Hatem 2021** | | | | | |
| --- | --- | --- | --- | --- | --- | --- |
| **Study identifier** |  | | | | | |
| **Reviewer** |  |  |  |  |  |  |
|  |  | | |  |  |  |
| **Biases** | **Issues to consider for judging overall rating of "Risk of bias"** | | | **Study Methods & Comments** | **Rating of reporting** | **Rating of "Risk of bias"** |
| Instructions to assess the risk of each potential bias: | These issues will guide your thinking and judgment about the overall risk of bias within each of the 6 domains. Some 'issues' may not be relevant to the specific study or the review research question. These issues are taken together to inform the overall judgment of potential bias for each of the 6 domains. | | | Provide comments or text exerpts in the white boxes below, as necessary, to facilitate the consensus process that will follow. | Click on each of the blue cells and choose from the drop down menu to rate the adequacy of reporting as yes, partial, no or unsure. | Click on the green cells; choose from the drop-down menu to rate potential risk of bias for each of the 6 domains as High, Moderate, or Low considering all relevant issues |
| **1. Study Participation** | **Goal: To judge the risk of selection bias (likelihood that relationship between *PF* and**  ***outcome* is different for participants and eligible non-participants).** | | |  |  |  |
| *Source of target population* | The source population or population of interest is adequately described for key characteristics (LIST). | | | Fragile families and child wellbeing longitudinal study: which follows 4,898 children between 1998-2000 and their unmarried mothers (these were oversampled by a ratio of 3 to 1). Adequate characteristics are provided: adolescent mental health, food insecurity, housing, mother mental health, child gender, race, health insurance, maternal and household characteristics (age, marital status, education, income, prison history, depression, anxiety) adolescent economic hardship experiences | Yes |  |
| *Method used to identify population* | The sampling frame and recruitment are adequately described, including methods to identify the sample  sufficient to limit potential bias (number and type used, e.g., referral patterns in health care) | | | Yes, the sample of children chosen are those who participated in home assessment of food insecurity when the child was age 3 and 5 years old. Participants recruited using stratified random sampling technique | Yes |  |
| *Recruitment period* | Period of recruitment is adequately described | | | Yes 1998-2000 | Yes |  |
| *Place of recruitment* | Place of recruitment (setting and geographic location) are adequately described | | | Yes across 20 major US cities | Yes |  |
| *Inclusion and exclusion criteria* | Inclusion and exclusion criteria are adequately described (e.g., including explicit diagnostic criteria or “zero time” description). | | | Yes | Yes |  |
| *Adequate study participation* | There is adequate participation in the study by eligible individuals | | | Yes a total of =2,626 in the total analytic sample | Yes |  |
| *Baseline characteristics* | The baseline study sample (i.e., individuals entering the study) is adequately described for key characteristics  (LIST). | | | Yes these are listed above. Excluded:  Those who did not participate in the year 15 interview  Mothers who did not participate in the year 9 mother interview  Families who were missing both year 3 and 5 in home assessments  Mothers who were not interviewed at year 3  Missing data on adolescent measure of anxiety and depressive symptoms and covariates | Yes |  |
| **Summary Study participation** | **The study sample represents the population of interest on key characteristics, sufficient to limit**  **potential bias of the observed relationship between PF and outcome.** | | | Low risk was concluded for study participants in terms of observing the relationship between HFI and the outcome. |  | Yes |
|  | | | | | | |
| **2. Study Attrition** | **Goal: To judge the risk of attrition bias (likelihood that relationship between *PF* and**  ***outcome* are different for completing and non-completing participants).** | | |  |  |  |
| *Proportion of baseline sample available*  *for analysis* | Response rate (i.e., proportion of study sample completing the study and providing outcome data) is adequate. | | | About half of the sample were excluded but this is still a large enough sample to gain meaningful results | Yes |  |
| *Attempts to collect information on*  *participants who dropped out* | Attempts to collect information on participants who dropped out of the study are described. | | | Details on dropouts were not provided | No |  |
| *Reasons and potential impact of*  *subjects lost to follow-up* | Reasons for loss to follow-up are provided. | | | N/A | N/A |  |
| *Outcome and prognostic factor information on those lost to follow-up* | Participants lost to follow-up are adequately described for key characteristics (LIST). | | | N/A | N/A |  |
|  | There are no important differences between key characteristics (LIST) and outcomes in participants who  completed the study and those who did not. | | | N/A | N/A |  |
| **Study Attrition Summary** | **Loss to follow-up (from baseline sample to study population analyzed) is not associated with key characteristics (i.e., the study data adequately represent the sample) sufficient to limit potential bias to the observed relationship between PF and outcome.** | | | The sample of data used did not account for loss to follow up or give information on the participants who were missing or excluded from the data analysis. This is ok as there was enough sample size to answer the specific question in the paper – the study was not specifically designed to answer this question and only a select sample of women were chosen to answer food insecurity/housing questions.  Where year 5 data were not available, year 3 data were used because of the correlational relationship between the two (it was hypothesized based on previous research that those who experienced FI in year 3 would also experience FI in year 5 so it seemed appropriate to use this). However, FI is cyclical in nature so this may not be the case and assumption so introduced bias into the study. Therefore, moderate bias is concluded. |  | moderate |
| 3. Prognostic Factor  Measurement | **Goal: To judge the risk of measurement bias related to how PF was measured (differential measurement of PF related to the level of outcome).** | | |  |  |  |
| Definition of the PF | **A clear definition or description of 'PF' is provided (e.g., including dose, level, duration of exposure, and clear**  **specification of the method of measurement).** | | | Yes, it is food insecurity and the method of measurement provided is the 18 item USDA questionnaire | Yes |  |
| Valid and Reliable Measurement of PF | **Method of PF measurement is adequately valid and reliable to limit misclassification bias (e.g., may include relevant outside sources of information on measurement properties, also characteristics, such as blind**  **measurement and limited reliance on recall).** | | | Yes it is a valid method of measurement | Yes |  |
|  | **Continuous variables are reported or appropriate cut-points (i.e., not data-dependent) are used.** | | | Cut offs based on affirmative responses – 0 to 2 affirmative responses (food secure) and 3 or more (food insecure) | Yes |  |
| Method and Setting of PF Measurement | **The method and setting of measurement of PF is the same for all study participants.** | | | Yes mothers were provided with the assessment | Yes |  |
| Proportion of data on PF available for  analysis | **Adequate proportion of the study sample has complete data for PF variable.** | | | Yes N=2626 | Yes |  |
| Method used for missing data | **Appropriate methods of imputation are used for missing 'PF' data.** | | | Yes where data is missing for example if the mother answers for year 5 and not year 3, then year 3 will be used for year 5. | Yes |  |
| PF Measurement Summary | **PF is adequately measured in study participants to sufficiently limit potential bias.** | | | Yes |  | Moderate |
|  | | | | | |  |
| **4. Outcome**  **Measurement** | | **Goal: To judge the risk of bias related to the measurement of outcome (differential measurement of outcome related to the baseline level of PF).** |  | |  |  |
| *Definition of the Outcome* | | A clear definition of outcome is provided, including duration of follow-up and level and extent of the outcome  construct. | Yes:  Depressive symptoms, anxiety symptoms, potential mediators (maternal depression and stress). The outcomes are defined by specific measurement scales and surveys (see the next bit) | | Yes |  |
| *Valid and Reliable Measurement of Outcome* | | The method of outcome measurement used is adequately valid and reliable to limit misclassification bias (e.g., may include relevant outside sources of information on measurement properties, also characteristics, such as blind measurement and confirmation of outcome with valid and reliable test). | Depressive symptoms: adolescent self reported using the 5 items of the Centers for Epidemiologic Studies Depression Scale  Anxiety symptoms: 6 items of the Brief symptoms inventory 18 item anxiety subscale  Maternal depression: 15 item composite international diagnostic interview short form at year 9  Parenting stress: Parenting stress inventory at year 9 | | Yes |  |
| *Method and Setting of Outcome*  *Measurement* | | The method and setting of outcome measurement is the same for all study participants. | Yes | | Yes |  |
| **Outcome Measurement Summary** | | ***Outcome of interest* is adequately measured in study participants to sufficiently limit potential bias.** | Yes | |  | Low |
|  | | | | | |  |
| **5. Study Confounding** | | **Goal: To judge the risk of bias due to confounding (i.e. the effect of PF is distorted by**  **another factor that is related to PF and outcome).** |  | |  |  |
| *Important Confounders Measured* | | All important confounders, including treatments (key variables in conceptual model: LIST), are measured. | Yes, confounders such as: child gender, health insurance at year 3, child race/ethnicity.  Maternal covariates at year 3: mothers age, marital status, education, employment, income compared to the poverty line, history of paternal incarceration, maternal anxiety and depression (due to generational pass of this) | | Yes |  |
| *Definition of the confounding factor* | | Clear definitions of the important confounders measured are provided (e.g., including dose, level, and duration  of exposures). | Yes, all confounders are described adequately. Education is measured by 3 categories (less than high school diploma, high school diploma and college), income is categorised based on the federal poverty line. | | Yes |  |
| *Valid and Reliable Measurement of Confounders* | | Measurement of all important confounders is adequately valid and reliable (e.g., may include relevant outside sources of information on measurement properties, also characteristics, such as blind measurement and limited  reliance on recall). | Yes: survey answers using mental health surveys, and then general questions about education/employment which are self-reported. | | Yes |  |
| *Method and Setting of Confounding*  *Measurement* | | The method and setting of confounding measurement are the same for all study participants. | Yes, these are self-reported using the questionnaires that are administered to everyone in the same way | | Yes |  |
| *Method used for missing data* | | Appropriate methods are used if imputation is used for missing confounder data. | Not reported not sure | | Not sure |  |
| *Appropriate Accounting for Confounding* | | Important potential confounders are accounted for in the study design (e.g., matching for key variables,  stratification, or initial assembly of comparable groups). | Yes all key variables, same as above. | | Yes |  |
|  |  | Important potential confounders are accounted for in the analysis (i.e., appropriate adjustment). | Yes statistical analysis adjusts for all relevant confounders. | | Yes |  |
| **Study Confounding Summary** | | **Important potential confounders are appropriately accounted for, limiting potential bias with respect to**  **the relationship between *PF* and *outcome* .** | All potential confounders are adequately included to limit bias. Low bias is concluded. | |  | low |
|  | | | | | |  |
| **6. Statistical Analysis**  **and Reporting** | | **Goal: To judge the risk of bias related to the statistical analysis and presentation of results.** |  | |  |  |
| *Presentation of analytical strategy* | | There is sufficient presentation of data to assess the adequacy of the analysis. | Yes descriptive statistics and key results are provided | | Yes |  |
| *Model development strategy* | | The strategy for model building (i.e., inclusion of variables in the statistical model) is appropriate and is based  on a conceptual framework or model. | Yes a conceptual framework is provided in the form of the Family Stress Model theoretical framework. This is what the inclusion of the variables in the analysis is based on. | | Yes |  |
|  |  | The selected statistical model is adequate for the design of the study. | Yes an SEM is chosen which is a method for mediation analysis | | Yes |  |
| *Reporting of results* | | There is no selective reporting of results. | None detected | | yes |  |
| **Statistical Analysis and Presentation**  **Summary** | | **The statistical analysis is appropriate for the design of the study, limiting potential for presentation of**  **invalid or spurious results.** | Low bias concluded. | |  | low |

| **Author and year of publication** | **Lohman et al., 2009** | | | | |
| --- | --- | --- | --- | --- | --- |
| **Study identifier** | Sarah Abraham | | | | |
| **Reviewer** |  |  |  |  |  |
|  |  | |  |  |  |
| **Biases** | **Issues to consider for judging overall rating of "Risk of bias"** | | **Study Methods & Comments** | **Rating of reporting** | **Rating of "Risk of bias"** |
| Instructions to assess the risk of each potential bias: | These issues will guide your thinking and judgment about the overall risk of bias within each of the 6 domains. Some 'issues' may not be relevant to the specific study or the review research question. These issues are taken together to inform the overall judgment of potential bias for each of the 6 domains. | | Provide comments or text exerpts in the white boxes below, as necessary, to facilitate the consensus process that will follow. | Click on each of the blue cells and choose from the drop down menu to rate the adequacy of reporting as yes, partial, no or unsure. | Click on the green cells; choose from the drop-down menu to rate potential risk of bias for each of the 6 domains as High, Moderate, or Low considering all relevant issues |
| **1. Study Participation** | **Goal: To judge the risk of selection bias (likelihood that relationship between *PF* and**  ***outcome* is different for participants and eligible non-participants).** | |  |  |  |
| *Source of target population* | The source population or population of interest is adequately described for key characteristics (LIST). | | Yes, the information provided is. The source of population is the The Three City study that was conducted in 1999. N=1011 adolescents aged 10-15 years old were analysed in this sample along with their mothers. |  |  |
| *Method used to identify population* | The sampling frame and recruitment are adequately described, including methods to identify the sample  sufficient to limit potential bias (number and type used, e.g., referral patterns in health care) | | The study uses a sample of participants from the Welfare Children and family a three city study 6-year longitudinal dataset.  The study used a household-based stratified sample survey of over 2,400 children and their mothers in low income neighborhoods of Boston, Chicago and San Antonio.  This article used data from the 1999 wave of interviews.  Households were randomly selected if they were in neighborhoods with poverty rates of at least 40%. A screener visited each home to see whether they were eligible for the study: whether there was a child aged 0-15 years old, whether the household had income less than double the poverty line, primary caregiver female and whether the household head was non-Hispanic white. Non-Hispanic black or Hispanic.  85% households were eligible and there was a response rate of 74%. | Yes |  |
| *Recruitment period* | Period of recruitment is adequately described | | 1999 | Yes |  |
| *Place of recruitment* | Place of recruitment (setting and geographic location) are adequately described | | In the home, families were visited by the recruiter for eligibility. | Yes |  |
| *Inclusion and exclusion criteria* | Inclusion and exclusion criteria are adequately described (e.g., including explicit diagnostic criteria or “zero time” description). | | Adolescents aged 10-15 years old were analyzed in this sample along with their mothers.  Families with incomes below twice the poverty line selected. White, Black and Hispanic head of the households chosen. | Yes |  |
| *Adequate study participation* | There is adequate participation in the study by eligible individuals | | Yes 74% response rate n=1011 | Yes |  |
| *Baseline characteristics* | The baseline study sample (i.e., individuals entering the study) is adequately described for key characteristics  (LIST). | | Yes. Characteristics provided (means, SD and percentages) for:  Adolescent weight status by CDC and IOTF cut offs,  Adolescent food insecurity,  Adolescent age, gender, race, TV watching time, disability that cause limitations, whether they were born with low birth weight, whether they are biologically related to their female caregiver, maternal BMI, maternal education, whether female caregiver is an immigrant, receiving TANF, food stamps, family income to needs ratio,  Individual stressors, maternal stressors, family stressors. | Yes |  |
| **Summary Study participation** | **The study sample represents the population of interest on key characteristics, sufficient to limit**  **potential bias of the observed relationship between PF and outcome.** | | Low bias concluded for study participation. |  | Low |
|  | | | | | |
| **2. Study Attrition** | **Goal: To judge the risk of attrition bias (likelihood that relationship between *PF* and**  ***outcome* are different for completing and non-completing participants).** | |  |  |  |
| *Proportion of baseline sample available*  *for analysis* | Response rate (i.e., proportion of study sample completing the study and providing outcome data) is adequate. | | All samples available for analysis. This was a secondary analysis of cross-sectional data. | Yes |  |
| *Attempts to collect information on*  *participants who dropped out* | Attempts to collect information on participants who dropped out of the study are described. | | N/A | N/A yes |  |
| *Reasons and potential impact of*  *subjects lost to follow-up* | Reasons for loss to follow-up are provided. | | N/A | N/A yes |  |
| *Outcome and prognostic factor information on those lost to follow-up* | Participants lost to follow-up are adequately described for key characteristics (LIST). | | N/A | N/A yes |  |
|  | There are no important differences between key characteristics (LIST) and outcomes in participants who  completed the study and those who did not. | | N/A |  |  |
| **Study Attrition Summary** | **Loss to follow-up (from baseline sample to study population analyzed) is not associated with key characteristics (i.e., the study data adequately represent the sample) sufficient to limit potential bias to the observed relationship between PF and outcome.** | | Loss to follow not applicable for this dataset as this is a sample taken from cross-sectional data. Low bias was detected as the participant inclusion/exclusion criteria provided was well justified. |  | Low |
|  | | | | | |
| **3. Prognostic Factor**  **Measurement** | | **Goal: To judge the risk of measurement bias related to how PF was measured (differential measurement of PF related to the level of outcome).** |  |  |  |
| *Definition of the PF* | | A clear definition or description of 'PF' is provided (e.g., including dose, level, duration of exposure, and clear  specification of the method of measurement). | Yes, it is food insecurity and takes three questions from the validated 18-item USDA food security questionnaire. These were mother-reported not adolescent-reported.  Questions:   1. At any time in the last 12 months did you cut the size of any of the adolescents meals because there wasn’t enough money for food? 2. At any time in the past 12 months was the adolescent hungry but you just couldn’t afford more food? 3. At any time in the past 12 months, did the adolescent skip a meal because there wasn’t enough money for food?   . | Yes |  |
| *Valid and Reliable Measurement of PF* | | Method of PF measurement is adequately valid and reliable to limit misclassification bias (e.g., may include relevant outside sources of information on measurement properties, also characteristics, such as blind  measurement and limited reliance on recall). | The same questions were asked in the current population survey for a sample of households similar to those used in this study and found good agreement between answers for both – not sure if this justifies no bias. | No |  |
|  |  | Continuous variables are reported or appropriate cut-points (i.e., not data-dependent) are used. | For weight status, the authors use the IOTF and CDC cut-off points in separate analyses. | Yes |  |
| *Method and Setting of PF Measurement* | | The method and setting of measurement of PF is the same for all study participants. | Yes | Yes |  |
| *Proportion of data on PF available for*  *analysis* | | Adequate proportion of the study sample has complete data for PF variable. | Yes | Yes |  |
| *Method used for missing data* | | Appropriate methods of imputation are used for missing 'PF' data. | N/A | Yes |  |
| **PF Measurement Summary** | | ***PF* is adequately measured in study participants to sufficiently limit potential bias.** | Low bias was concluded for the study participants. |  | Low |
|  | | | | | |
| **4. Outcome**  **Measurement** | | **Goal: To judge the risk of bias related to the measurement of outcome (differential measurement of outcome related to the baseline level of PF).** |  |  |  |
| *Definition of the Outcome* | | A clear definition of outcome is provided, including duration of follow-up and level and extent of the outcome  construct. | Stressors are defined as external factors that may cause a stress response. Individual, maternal and family stressors indices were created. Each of these indices were derived from dichotomous variables.  Individual stressors index : five measured used: (i) self-reported academic achievements, (ii) maternal report of lack of future orientation for the adolescent, (iii) 17-item assessment of drugs and alcohol, (iv and v) child behavioural checklist for internalizing and externalizing adolescent behaviours.  Maternal stressor index: Six constructs used to assess maternal stressors. (i) value of 1 assigned if mother unemployed or worked less than 20 hours per week or worked more than 60 hours per week, (ii) value of 1 assigned if work was limited due to a disability, (iii) completion of the Rosenburg self-esteem scale, (iv) completion of the 18-item Brief symptom inventory, (v) value assigned to represent general health ranging from excellent to poor (vi) four-item social support questionnaire.  Family stressor index: 12 indicators to define family stressors. Indicators included: whether the mother owned her house and had housing problems such as problems with plumbing, questions on family routines (eating together, how often mother has family time, children having the same bedtime everynight, cognitive stimulation (13 item), parental monitoring ( sum of 7 items), mother-child activities (sum of 3 items), father involvement (sum of 7 items), lack of financial strain (sum of 5 items), any report of domestic violence for the mother (sum of 12 items). Family structure (mother married or not – 1 was assigned if not married), and whether mother had no health insurance (1 assigned if no health insurance and 1 if adolescent had no health insurance). | Yes |  |
| *Valid and Reliable Measurement of Outcome* | | The method of outcome measurement used is adequately valid and reliable to limit misclassification bias (e.g., may include relevant outside sources of information on measurement properties, also characteristics, such as blind measurement and confirmation of outcome with valid and reliable test). | Yes, all validated sources of outcome measures were used. | Yes |  |
| *Method and Setting of Outcome*  *Measurement* | | The method and setting of outcome measurement is the same for all study participants. | Yes BMI was measured by a trained personnel in the adolescents home | Yes |  |
| **Outcome Measurement Summary** | | ***Outcome of interest* is adequately measured in study participants to sufficiently limit potential bias.** | All outcomes of interest were adequately measured in study participants which concluded low bias. |  | Low |
|  | | | | | |
| **5. Study Confounding** | | **Goal: To judge the risk of bias due to confounding (i.e. the effect of PF is distorted by**  **another factor that is related to PF and outcome).** |  |  |  |
| *Important Confounders Measured* | | All important confounders, including treatments (key variables in conceptual model: LIST), are measured. | Yes. Age, household income. Race/ethnicity, television viewing, limitations due to disability, low birth weight, maternal specific: are biological mother, if she is an immigrant, currently receiving assistance financially or food stamps, education level, maternal bmi and household income ratio | Yes |  |
| *Definition of the confounding factor* | | Clear definitions of the important confounders measured are provided (e.g., including dose, level, and duration  of exposures). | Yes all were appropriately defined and are self-explanatory. | Yes |  |
| *Valid and Reliable Measurement of Confounders* | | Measurement of all important confounders is adequately valid and reliable (e.g., may include relevant outside sources of information on measurement properties, also characteristics, such as blind measurement and limited  reliance on recall). | The measurements were self-reported by the mother. | Yes |  |
| *Method and Setting of Confounding*  *Measurement* | | The method and setting of confounding measurement are the same for all study participants. | Yes all had in home interviews led by trained field investigators. | Not sure |  |
| *Method used for missing data* | | Appropriate methods are used if imputation is used for missing confounder data. | N/A | N/A |  |
| *Appropriate Accounting for Confounding* | | Important potential confounders are accounted for in the study design (e.g., matching for key variables,  stratification, or initial assembly of comparable groups). | Yes | Yes |  |
|  |  | Important potential confounders are accounted for in the analysis (i.e., appropriate adjustment). | Yes, as covariates in the statistical analyses | Yes |  |
| **Study Confounding Summary** | | **Important potential confounders are appropriately accounted for, limiting potential bias with respect to**  **the relationship between *PF* and *outcome* .** | Low bias was concluded. |  | Low |
|  | | | | | |
| **6. Statistical Analysis**  **and Reporting** | | **Goal: To judge the risk of bias related to the statistical analysis and presentation of results.** |  |  |  |
| *Presentation of analytical strategy* | | There is sufficient presentation of data to assess the adequacy of the analysis. | Yes | Yes |  |
| *Model development strategy* | | The strategy for model building (i.e., inclusion of variables in the statistical model) is appropriate and is based  on a conceptual framework or model. | Series of logistic regression models used to assess association of obesity/overweight. Two models presented:   1. Food insecurity and the stressor indices estimated overweight using CDC and IOTF classifications of overweight 2. Food insecurity, the stressor indices and the interaction of these indices with food insecurity   The analysis was based on previous literature. | Yes |  |
|  |  | The selected statistical model is adequate for the design of the study. | Yes | Yes |  |
| *Reporting of results* | | There is no selective reporting of results. | No | Yes |  |
| **Statistical Analysis and Presentation**  **Summary** | | **The statistical analysis is appropriate for the design of the study, limiting potential for presentation of**  **invalid or spurious results.** | Low bias was concluded. |  | Low |

| **Author and year of publication** | **Marcal 2022** | | | |
| --- | --- | --- | --- | --- |
| **Study identifier** |  | | | |
| **Reviewer** |  |  |  |  |
|  |  |  |  |  |
| **Biases** | **Issues to consider for judging overall rating of "Risk of bias"** | **Study Methods & Comments** | **Rating of reporting** | **Rating of "Risk of bias"** |
| Instructions to assess the risk of each potential bias: | These issues will guide your thinking and judgment about the overall risk of bias within each of the 6 domains. Some 'issues' may not be relevant to the specific study or the review research question. These issues are taken together to inform the overall judgment of potential bias for each of the 6 domains. | Provide comments or text exerpts in the white boxes below, as necessary, to facilitate the consensus process that will follow. | Click on each of the blue cells and choose from the drop down menu to rate the adequacy of reporting as yes, partial, no or unsure. | Click on the green cells; choose from the drop-down menu to rate potential risk of bias for each of the 6 domains as High, Moderate, or Low considering all relevant issues |
| **1. Study Participation** | **Goal: To judge the risk of selection bias (likelihood that relationship between *PF* and**  ***outcome* is different for participants and eligible non-participants).** |  |  |  |
| *Source of target population* | The source population or population of interest is adequately described for key characteristics (LIST). | Fragile families and child wellbeing longitudinal study: which follows 4,898 children between 1998-2000 and their unmarried mothers (these were oversampled by a ratio of 3 to 1). Adequate characteristics are provided: adolescent mental health, food insecurity, housing, mother mental health, child gender, race, health insurance, maternal and household characteristics (age, marital status, education, income, prison history, depression, anxiety) adolescent economic hardship experiences | Yes |  |
| *Method used to identify population* | The sampling frame and recruitment are adequately described, including methods to identify the sample  sufficient to limit potential bias (number and type used, e.g., referral patterns in health care) | The study used data from the baseline and year 5-15 interviews. Sampled randomly from stratified random sampling algorithm | Yes |  |
| *Recruitment period* | Period of recruitment is adequately described | Yes 1998-2000 | Yes |  |
| *Place of recruitment* | Place of recruitment (setting and geographic location) are adequately described | Yes across 20 major US cities | Yes |  |
| *Inclusion and exclusion criteria* | Inclusion and exclusion criteria are adequately described (e.g., including explicit diagnostic criteria or “zero time” description). | Yes | Yes |  |
| *Adequate study participation* | There is adequate participation in the study by eligible individuals | Yes a total of =2,454 in the total analytic sample | Yes |  |
| *Baseline characteristics* | The baseline study sample (i.e., individuals entering the study) is adequately described for key characteristics  (LIST). | Yes. Analytic sample included families in which the study focal child lived with the biological mother at least half of the time for the Year 5 and Year 9 interviews | Yes |  |
| **Summary Study participation** | **The study sample represents the population of interest on key characteristics, sufficient to limit**  **potential bias of the observed relationship between PF and outcome.** | Low bias was concluded for the participants included. |  | low |
|  | | | | |
| **2. Study Attrition** | **Goal: To judge the risk of attrition bias (likelihood that relationship between *PF* and**  ***outcome* are different for completing and non-completing participants).** |  |  |  |
| *Proportion of baseline sample available*  *for analysis* | Response rate (i.e., proportion of study sample completing the study and providing outcome data) is adequate. | About half of the sample were excluded but this is was still considered large enough sample to gain meaningful results | Yes |  |
| *Attempts to collect information on*  *participants who dropped out* | Attempts to collect information on participants who dropped out of the study are described. | There was very little missing data in the survey | Yes |  |
| *Reasons and potential impact of*  *subjects lost to follow-up* | Reasons for loss to follow-up are provided. | No | No |  |
| *Outcome and prognostic factor information on those lost to follow-up* | Participants lost to follow-up are adequately described for key characteristics (LIST). | No | No |  |
|  | There are no important differences between key characteristics (LIST) and outcomes in participants who  completed the study and those who did not. | Attrition analysis showed no difference in the missingness between outcomes by demographics | Yes |  |
| **Study Attrition Summary** | **Loss to follow-up (from baseline sample to study population analyzed) is not associated with key characteristics (i.e., the study data adequately represent the sample) sufficient to limit potential bias to the observed relationship between PF and outcome.** | Moderate bias was concluded as those from the most vulnerable or hard-to-reach groups could have been excluded from the sample. |  | Moderate |
|  |  |  |  |  |
|  | | | | |

| **3. Prognostic Factor**  **Measurement** | **Goal: To judge the risk of measurement bias related to how PF was measured (differential measurement of PF related to the level of outcome).** |  |  |  |
| --- | --- | --- | --- | --- |
| *Definition of the PF* | A clear definition or description of 'PF' is provided (e.g., including dose, level, duration of exposure, and clear  specification of the method of measurement). | Yes food insecurity was defined by the USDA definition of HFI. | Yes |  |
| *Valid and Reliable Measurement of PF* | Method of PF measurement is adequately valid and reliable to limit misclassification bias (e.g., may include relevant outside sources of information on measurement properties, also characteristics, such as blind  measurement and limited reliance on recall). | Yes, it is a valid method of measurement using a 10-item scale by the USDA | Yes |  |
|  | Continuous variables are reported or appropriate cut-points (i.e., not data-dependent) are used. | Food insecurity was measured on a two point scale (food insecure and food secure) the cut offs are not defined in the paper | Yes |  |
| *Method and Setting of PF Measurement* | The method and setting of measurement of PF is the same for all study participants. | Yes mothers were provided with the assessment | Yes |  |
| *Proportion of data on PF available for*  *analysis* | Adequate proportion of the study sample has complete data for PF variable. | Yes N=2454 | Yes |  |
| *Method used for missing data* | Appropriate methods of imputation are used for missing 'PF' data. | Yes, missing data was dealt with using multiple imputation by chained equations with predictive mean matching. The missing variable were predicted using the other variables as dependent variables. | Yes |  |
| **PF Measurement Summary** | ***PF* is adequately measured in study participants to sufficiently limit potential bias.** | Yes |  | low |
|  | | | | |
| **4. Outcome**  **Measurement** | **Goal: To judge the risk of bias related to the measurement of outcome (differential measurement of outcome related to the baseline level of PF).** | Low bias was concluded as authors used a validated questionnaire for HFI and also addressed missing data using multiple imputation. |  |  |
| *Definition of the Outcome* | A clear definition of outcome is provided, including duration of follow-up and level and extent of the outcome  construct. | Yes:  Adolescent aggressive behaviour was defined as disruptive and violent  Adolescent depressive behaviour was defined by symptoms of depression displayed with the youth  Parenting stress: stress of caregiving for the child when the child was aged 9 | Yes |  |
| *Valid and Reliable Measurement of Outcome* | The method of outcome measurement used is adequately valid and reliable to limit misclassification bias (e.g., may include relevant outside sources of information on measurement properties, also characteristics, such as blind measurement and confirmation of outcome with valid and reliable test). | Depressive symptoms: mother reported behaviour at age 15 of the child using the Child Behaviour Checklist (CBCL)  Aggressive behaviour: mother reported CBCL  Anxiety symptoms: 6 items of the Brief symptoms inventory 18 item anxiety subscale  Parenting stress: items from the Child Development Supplement of the Panel study of income dynamics and the parent stress inventory | Yes |  |
| *Method and Setting of Outcome*  *Measurement* | The method and setting of outcome measurement is the same for all study participants. | Yes | Yes |  |
| **Outcome Measurement Summary** | ***Outcome of interest* is adequately measured in study participants to sufficiently limit potential bias.** | Yes all outcome measures were reported using validated questionnaires and low bias was concluded. |  | Low |
|  | | | | |
| **5. Study Confounding** | **Goal: To judge the risk of bias due to confounding (i.e. the effect of PF is distorted by**  **another factor that is related to PF and outcome).** |  |  |  |
| *Important Confounders Measured* | All important confounders, including treatments (key variables in conceptual model: LIST), are measured. | mothers age, mothers race/ethnicity, mothers highest level of education, household income, mothers’ marital status, instrumental support, child gender, household income, family history of mental disorder and year 5 behaviour problems in the corresponding domains | yes |  |
| *Definition of the confounding factor* | Clear definitions of the important confounders measured are provided (e.g., including dose, level, and duration  of exposures). | Yes, all were self-explanatory, and definitions were also provided. | Yes |  |
| *Valid and Reliable Measurement of Confounders* | Measurement of all important confounders is adequately valid and reliable (e.g., may include relevant outside sources of information on measurement properties, also characteristics, such as blind measurement and limited  reliance on recall). | Yes: survey answers using mental health surveys, and then general questions about education/employment | Yes |  |
| *Method and Setting of Confounding*  *Measurement* | The method and setting of confounding measurement are the same for all study participants. | Yes these are self-reported using the questionnaires that are administered to everyone in the same way | yes |  |
| *Method used for missing data* | Appropriate methods are used if imputation is used for missing confounder data. | Multiple imputation methods | Yes |  |
| *Appropriate Accounting for Confounding* | Important potential confounders are accounted for in the study design (e.g., matching for key variables,  stratification, or initial assembly of comparable groups). | Yes for pathways to parenting stress: mothers age, mothers race/ethnicity, mothers highest level of education, household income, mothers’ marital status, instrumental support.  For pathways to adolescent behaviour problems: controlled for child gender, household income, family history of mental disorder and year 5 behaviour problems in the corresponding domains | Yes |  |
|  | Important potential confounders are accounted for in the analysis (i.e., appropriate adjustment). | Yes statistical analysis adjusted for covariates | Yes |  |
| **Study Confounding Summary** | **Important potential confounders are appropriately accounted for, limiting potential bias with respect to**  **the relationship between *PF* and *outcome* .** | Yes low bias was concluded for all confounder variables within the anaysis |  | low |
|  | | | | |
| **6. Statistical Analysis**  **and Reporting** | **Goal: To judge the risk of bias related to the statistical analysis and presentation of results.** |  |  |  |
| *Presentation of analytical strategy* | There is sufficient presentation of data to assess the adequacy of the analysis. | Yes data was presented for | Yes |  |
| *Model development strategy* | The strategy for model building (i.e., inclusion of variables in the statistical model) is appropriate and is based  on a conceptual framework or model. | Yes | Yes |  |
|  | The selected statistical model is adequate for the design of the study. | Yes | Yes |  |
| *Reporting of results* | There is no selective reporting of results. | No | Yes |  |
| **Statistical Analysis and Presentation**  **Summary** | **The statistical analysis is appropriate for the design of the study, limiting potential for presentation of**  **invalid or spurious results.** | Yes |  | low |

| **Author and year of publication** | **Willis and Fitzpatrick (2016)** | | | | | |
| --- | --- | --- | --- | --- | --- | --- |
| **Study identifier** |  | | | | | |
| **Reviewer** |  |  |  |  |  |  |
|  |  |  | |  | |  |
| **Biases** | **Issues to consider for judging overall rating of "Risk of bias"** | **Study Methods & Comments** | | **Rating of reporting** | | **Rating of "Risk of bias"** |
| Instructions to assess the risk of each potential bias: | These issues will guide your thinking and judgment about the overall risk of bias within each of the 6 domains. Some 'issues' may not be relevant to the specific study or the review research question. These issues are taken together to inform the overall judgment of potential bias for each of the 6 domains. | Provide comments or text exerpts in the white boxes below, as necessary, to facilitate the consensus process that will follow. | | Click on each of the blue cells and choose from the drop down menu to rate the adequacy of reporting as yes, partial, no or unsure. | | Click on the green cells; choose from the drop-down menu to rate potential risk of bias for each of the 6 domains as High, Moderate, or Low considering all relevant issues |
| **1. Study Participation** | **Goal: To judge the risk of selection bias (likelihood that relationship between *PF* and**  ***outcome* is different for participants and eligible non-participants).** |  | |  | |  |
| *Source of target population* | The source population or population of interest is adequately described for key characteristics (LIST). | Cross sectional data from 5^th^ and 7^th^ graders in a middle school in North west Arkansas administered in late September of 2012: Sample demographics of weight status, age, sex, race, ethnicity, psychosocial resources and risk. No maternal or parental information provided. Demographic provided in code (e.g. Hispanic ethnicity =1) | | Yes | |  |
| *Method used to identify population* | The sampling frame and recruitment are adequately described, including methods to identify the sample  sufficient to limit potential bias (number and type used, e.g., referral patterns in health care) | The study initially enrolled 361 students. Not sure how the school was selected. All fifth, sixth and seventh grade students were included in the sampling frame | | Yes | |  |
| *Recruitment period* | Period of recruitment is adequately described | Yes September 2012 | | Yes | |  |
| *Place of recruitment* | Place of recruitment (setting and geographic location) are adequately described | Yes school setting in Arkansas. | | Yes | |  |
| *Inclusion and exclusion criteria* | Inclusion and exclusion criteria are adequately described (e.g., including explicit diagnostic criteria or “zero time” description). | Yes: those who did not complete the survey | | Yes | |  |
| *Adequate study participation* | There is adequate participation in the study by eligible individuals | Yes a total of =2,454 in the total analytic sample | | Yes | |  |
| *Baseline characteristics* | The baseline study sample (i.e., individuals entering the study) is adequately described for key characteristics  (LIST). | Yes fifth, sixth and seventh graders who completed the survey. Description not given in detail | | Yes | |  |
| **Summary Study participation** | **The study sample represents the population of interest on key characteristics, sufficient to limit**  **potential bias of the observed relationship between PF and outcome.** | Description of students not provided in much detail, moderate bias was concluded. | |  | | moderate |
|  | | | | | | |
| **2. Study Attrition** | **Goal: To judge the risk of attrition bias (likelihood that relationship between *PF* and**  ***outcome* are different for completing and non-completing participants).** |  | |  | |  |
| *Proportion of baseline sample available*  *for analysis* | Response rate (i.e., proportion of study sample completing the study and providing outcome data) is adequate. | Yes, out of 361 students N=324 students completed the survey and were included in the study | | Yes | |  |
| *Attempts to collect information on*  *participants who dropped out* | Attempts to collect information on participants who dropped out of the study are described. | There was very little missing data in the survey. The authors did collect data to understand the reason for the drop out. | | Yes | |  |
| *Reasons and potential impact of*  *subjects lost to follow-up* | Reasons for loss to follow-up are provided. | Nearly all those who did not complete survey were missing due to absence due to illness or had another form of excused absence. Three students chose not to complete the survey | | Yes | |  |
| *Outcome and prognostic factor information on those lost to follow-up* | Participants lost to follow-up are adequately described for key characteristics (LIST). | Yes | | Yes | |  |
|  | There are no important differences between key characteristics (LIST) and outcomes in participants who  completed the study and those who did not. | Not sure this was not reported | | Not sure | |  |
| **Study Attrition Summary** | **Loss to follow-up (from baseline sample to study population analyzed) is not associated with key characteristics (i.e., the study data adequately represent the sample) sufficient to limit potential bias to the observed relationship between PF and outcome.** | Low risk of bias was concluded as there was some indication of why the missingness occurred. | |  | | low |
|  |  |  | |  | |  |
|  | | | | | | |
| **3. Prognostic Factor**  **Measurement** | **Goal: To judge the risk of measurement bias related to how PF was measured (differential measurement of PF related to the level of outcome).** |  |  | |  | |
| *Definition of the PF* | A clear definition or description of 'PF' is provided (e.g., including dose, level, duration of exposure, and clear  specification of the method of measurement). | The definition of HFI was taken from the USDA. | Yes | |  | |
| *Valid and Reliable Measurement of PF* | Method of PF measurement is adequately valid and reliable to limit misclassification bias (e.g., may include relevant outside sources of information on measurement properties, also characteristics, such as blind  measurement and limited reliance on recall). | Yes, it is a valid method of measurement from the USDA HFI survey was used. | yes | |  | |
|  | Continuous variables are reported or appropriate cut-points (i.e., not data-dependent) are used. | A composite food insecurity scale was computed using the data ranging from 0 to 10. Authors deemed the scale reliable based on the Cronbach’s alpha of 0.84. – used the data cut off values not provided | Yes | |  | |
| *Method and Setting of PF Measurement* | The method and setting of measurement of PF is the same for all study participants. | Yes mothers were told to complete the survey. | yes | |  | |
| *Proportion of data on PF available for*  *analysis* | Adequate proportion of the study sample has complete data for PF variable. | Yes N=2454 | Yes | |  | |
| *Method used for missing data* | Appropriate methods of imputation are used for missing 'PF' data. | Yes missing data was dealt with using multiple imputation by chained equations with predictive mean matching. The missing variable were predicted using the other variables as dependent variables. | Yes | |  | |
| **PF Measurement Summary** | ***PF* is adequately measured in study participants to sufficiently limit potential bias.** | There was some bias detected as missing data was imputed depending on the values that were present in the data and a lack of information was provided for the demographics of the participants who were missing. |  | | Moderate | |
|  | | | | | | |
| **4. Outcome**  **Measurement** | **Goal: To judge the risk of bias related to the measurement of outcome (differential measurement of outcome related to the baseline level of PF).** |  |  | |  | |
| *Definition of the Outcome* | A clear definition of outcome is provided, including duration of follow-up and level and extent of the outcome  construct. | Yes:  Weight status: categories determined by the CDC  Poverty: defined by whether children pay for their school lunch or receive a free or reduced-price lunch  Depression: symptoms of depression  Social capital: about the child’s close friends/ acquaintances  Self esteem: how children perceive themselves in comparison to others  Perceived social class/status: how children perceive their household socioeconomic status and economic situation at home | yes | |  | |
| *Valid and Reliable Measurement of Outcome* | The method of outcome measurement used is adequately valid and reliable to limit misclassification bias (e.g., may include relevant outside sources of information on measurement properties, also characteristics, such as blind measurement and confirmation of outcome with valid and reliable test). | Depression: shortened version of the Center for Epidemiological studies for depression scale  Poverty: using proxy measure of free school meals or reduced school lunches  Depression: 8 item measure of the original 20 item center for epidemiological studies for depression scale (CES-D scale)  Social capital: numerical response, answering the question “how many close friends do you have?” – children were given cues on how to distinguish between close friends, friends and acquantainces – there is no more information about these cues  Self esteem: The 10 item Rosenberg’s self esteem index used as an indicator of psychological measure  Perceived social class: questions such as “thinking about the money your family has and the things your family owns, would you think of your family as…” and then a chose of class for example (upper, middle, lower class”). How these classes are defined and explained to the adolescents is not provided. | yes | |  | |
| *Method and Setting of Outcome*  *Measurement* | The method and setting of outcome measurement is the same for all study participants. | Yes | yes | |  | |
| **Outcome Measurement Summary** | ***Outcome of interest* is adequately measured in study participants to sufficiently limit potential bias.** | Yes: however, some definitions such as of the class system and the basis of questions and their validity is not provided |  | | Moderate | |
|  | | | | | | |
| **5. Study Confounding** | **Goal: To judge the risk of bias due to confounding (i.e. the effect of PF is distorted by**  **another factor that is related to PF and outcome).** |  |  | |  | |
| *Important Confounders Measured* | All important confounders, including treatments (key variables in conceptual model: LIST), are measured. | Sex and ethnicity the only controls that were deemed meaningful. Age was not used, as this was an age-specific sample. | Yes | |  | |
| *Definition of the confounding factor* | Clear definitions of the important confounders measured are provided (e.g., including dose, level, and duration  of exposures). | Yes | Yes | |  | |
| *Valid and Reliable Measurement of Confounders* | Measurement of all important confounders is adequately valid and reliable (e.g., may include relevant outside sources of information on measurement properties, also characteristics, such as blind measurement and limited  reliance on recall). | Yes, self-reported by adolescents | Yes | |  | |
| *Method and Setting of Confounding*  *Measurement* | The method and setting of confounding measurement are the same for all study participants. | Yes these are self reported using the questionnaires that are administered to everyone in the same way | Yes | |  | |
| *Method used for missing data* | Appropriate methods are used if imputation is used for missing confounder data. | All cases with missing key data were excluded | yes | |  | |
| *Appropriate Accounting for Confounding* | Important potential confounders are accounted for in the study design (e.g., matching for key variables,  stratification, or initial assembly of comparable groups). | No – not household information is provided | no | |  | |
|  | Important potential confounders are accounted for in the analysis (i.e., appropriate adjustment). | No confounders were limited to only age and ethnicity, other markers such as SES were not included. | yes | |  | |
| **Study Confounding Summary** | **Important potential confounders are appropriately accounted for, limiting potential bias with respect to**  **the relationship between *PF* and *outcome* .** | Moderate bias was concluded because not confounders used to adjust the analysis were limited. |  | | moderate | |
|  | | | | | | |
| **6. Statistical Analysis**  **and Reporting** | **Goal: To judge the risk of bias related to the statistical analysis and presentation of results.** |  |  | |  | |
| *Presentation of analytical strategy* | There is sufficient presentation of data to assess the adequacy of the analysis. | Yes | Yes | |  | |
| *Model development strategy* | The strategy for model building (i.e., inclusion of variables in the statistical model) is appropriate and is based  on a conceptual framework or model. | The study cited the previous literature as justification for the model, but it was unclear why key confounders were excluded. | Unsure | |  | |
|  | The selected statistical model is adequate for the design of the study. | Yes | Yes | |  | |
| *Reporting of results* | There is no selective reporting of results. | No | Yes | |  | |
| **Statistical Analysis and Presentation**  **Summary** | **The statistical analysis is appropriate for the design of the study, limiting potential for presentation of**  **invalid or spurious results.** | Moderate bias was concluded. |  | | Moderate | |
